# 3D Printing of Microenvironment‐Specific Bioinspired and Exosome‐Reinforced Hydrogel Scaffolds for Efficient Cartilage and Subchondral Bone Regeneration

**DOI:** 10.1002/advs.202303650

**Published:** 2023-07-09

**Authors:** Qi Li, Huilei Yu, Fengyuan Zhao, Chenxi Cao, Tong Wu, Yifei Fan, Yingfang Ao, Xiaoqing Hu

**Affiliations:** ^1^ Department of Sports Medicine Institute of Sports Medicine of Peking University Beijing Key Laboratory of Sports Injuries Peking University Third Hospital Beijing 100191 China; ^2^ Center of Foot and Ankle Surgery Beijing Tongren Hospital Capital Medical University Beijing 100730 China

**Keywords:** 3D printing, cartilage and bone regeneration, decellularized extracellular matrix, exosomes, scaffolds

## Abstract

In clinical practice, repairing osteochondral defects presents a challenge due to the varying biological properties of articular cartilages and subchondral bones. Thus, elucidating how spatial microenvironment‐specific biomimetic scaffolds can be used to simultaneously regenerate osteochondral tissue is an important research topic. Herein, a novel bioinspired double‐network hydrogel scaffold produced via 3D printing with tissue‐specific decellularized extracellular matrix (dECM) and human adipose mesenchymal stem cell (MSC)‐derived exosomes is described. The bionic hydrogel scaffolds promote rat bone marrow MSC attachment, spread, migration, proliferation, and chondrogenic and osteogenic differentiation in vitro, as determined based on the sustained release of bioactive exosomes. Furthermore, the 3D‐printed microenvironment‐specific heterogeneous bilayer scaffolds efficiently accelerate the simultaneous regeneration of cartilage and subchondral bone tissues in a rat preclinical model. In conclusion, 3D dECM‐based microenvironment‐specific biomimetics encapsulated with bioactive exosomes can serve as a novel cell‐free recipe for stem cell therapy when treating injured or degenerative joints. This strategy provides a promising platform for complex zonal tissue regeneration whilst holding attractive clinical translation potential.

## Graphical abstract

A novel tissue‐specific microenvironment biomimetic and exosome‐reinforced hydrogel scaffold for complex heterogeneous tissue regeneration is designed. The 3D‐printed bioinspired hydrogel scaffold can efficiently promote mesenchymal stem cell lineage‐specific differentiation and osteochondral regeneration in a rat model. The strategy provides a promising platform for physiological regeneration of complex zonal tissue and holds attractive clinical translation potential.

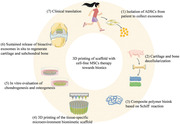

## 1 Introduction

Articular chondral lesions are present in >60% of patients that undergo arthroscopy.^[^ ^1^, ^2^ ^]^ Cartilage has a limited self‐healing capacity due to its avascular nature and low cellularity. Articular cartilage tissues connect articular surfaces to subchondral bones. In clinical situations, cartilage damage frequently extends deep into the associated subchondral bone and thus causes osteochondral (OC) defects,^[^ ^3^ ^]^ resulting in severe pain, motor disturbances, and degenerative joint diseases that affect millions of people worldwide.^[^ ^4^, ^5^ ^]^ Current clinical approaches, such as introducing microfractures and autologous chondrocyte implantations, are applicable for the restoration of joint surface cartilage, but are unsatisfactory and unsuitable for treating OC defects.^[^ ^6^ ^]^ Cartilage and subchondral bone tissues have different biological lineages and distinct properties, whilst effectively repairing entire OC defects remains a significant challenge. Recently, progress has been made in tissue‐engineering technology for repairing OC defects using bioceramics, metals, and polymeric biomaterials.^[^ ^7^, ^8^, ^9^ ^]^ However, homogeneous scaffolds do not have the same complexity as an OC matrix.^[^ ^10^ ^]^ Consequently, the fabrication of a spatially biomimetic bilayer scaffold that possesses chondrogenic and osteogenic microenvironments similar to those of native OC tissues represents a promising, yet challenging strategy for the simultaneous regeneration of cartilage and subchondral bone tissues.

The extracellular matrix (ECM) constitutes an important microenvironment for cell proliferation, differentiation, and the maintenance of physiological activities. Ideally, biomimetic scaffolds for tissue engineering should provide this kind of microenvironment to replicate the native features of ECM. Decellularized ECM (dECM) is one of the best candidates for fully emulating the intricacies of natural ECM.^[^ ^11^, ^12^ ^]^ The results of previous studies have shown that dECMs can facilitate mesenchymal stem cell (MSC) recruitment and differentiation, modulate macrophage polarization, and promote tissue repair without biological toxicity.^[^ ^13^, ^14^, ^15^, ^16^ ^]^ Although dECM scaffolds derived from cartilage (DCM), bone (DBM), peritoneum, derma, and small intestinal submucosa are used for cartilage tissue restoration after injury,^[^ ^17^, ^18^, ^19^ ^]^ dECMs originating from different tissues are unlikely to be optimal for regenerating the complex OC region of the joints. This premise has been strengthened by recent evidence suggesting that dECMs derived from a specific region, tissue, or organ can direct the differentiation of progenitor cells toward the phenotype of the corresponding original lineage.^[^ ^20^, ^21^, ^22^, ^23^ ^]^ Therefore, the ideal scenario for OC‐region regeneration would be to use resident bioactive cells to reconstruct a natural spatial microenvironment similar to the site‐specific parent tissues, namely DCM for cartilage remodeling and DBM for subchondral bone reconstruction.^[^ ^24^ ^]^ Difficulties are associated with fabricating stratified OC scaffolds using traditional tissue engineering techniques, such as freeze‐drying. 3D printing has recently emerged as an ideal strategy for accurately constructing tissues or organs with complex spatial structures, although printing pure dECM hydrogels could also be challenging.^[^ ^25^ ^]^ For instance, pure dECM hydrogels show weak mechanical properties that are insufficient for maintaining a multilayer (layer‐by‐layer) scaffold. In addition, the relatively rapid degradation profile of pure dECM hydrogels are not conducive for the longer process of tissue regeneration,^[^ ^26^ ^]^ which necessitates introducing additional biomaterials, including gelatin, hyaluronic acid (HA), alginate, collagen, and their derivatives (or hybrids thereof) to make better use of dECM hydrogels in 3D printing.

MSC therapy presents a promising strategy for cartilage and bone disease therapy, although its clinical translation and application have several limitations, such as immunosuppression and undefined differentiation lineages in vivo.^[^ ^27^ ^]^ Exosomes (also known as small extracellular vesicles) have a diameter of ≈40–160 nm and can efficiently deliver bioactive cargoes molecules (proteins, metabolites, and nucleic acids) into recipient cells, effectively regulating their biological responses.^[^ ^28^ ^]^ As natural nanoparticles or drug‐delivery vehicles that restore injured tissues,^[^ ^29^, ^30^, ^31^, ^32^ ^]^ exosomes are highly promising for next‐generation nanomedicine approaches.^[^ ^33^ ^]^ Previous findings have shown that multiple injections of MSC‐derived exosomes can accelerate cartilage regeneration,^[^ ^34^, ^35^ ^]^ suggesting that biomaterials based on bioactive exosomes merit development and have broad prospects for clinical translation.^[^ ^36^, ^37^ ^]^ Therefore, several researchers have developed scaffolds with encapsulated exosomes or coated with exosomes that can promote cartilage or bone regeneration.^[^ ^38^, ^39^ ^]^ To our knowledge, no previous reports have described a combined strategy involving the sustained release of exosomes from spatial‐biological microenvironment biomimetics for the efficient and simultaneous repair of cartilage and subchondral bone defects.

**Figure 1:**
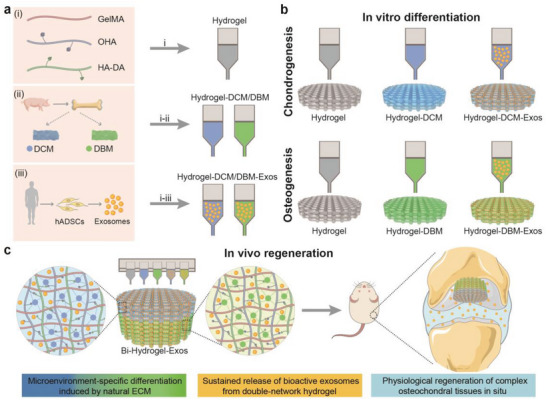
Schematic illustration of 3D printing of microenvironment‐specific biomimetic hydrogel scaffolds for the repairing of osteochondral (OC) defects. a), i) Methacrylated gelatin (GelMA), oxidative hyaluronic acid (OHA), and dopamine‐conjugated HA (HA‐DA) are synthesized and prepared for Hydrogel bioink. ii) Decellularized extracellular matrix (dECM) of cartilage (DCM) and bone (DBM) from porcine are incorporated into Hydrogel bioink to form microenvironment‐specific biomimetic Hydrogel‐DCM and Hydrogel‐DBM bioink, respectively. iii) Exosomes isolated from human adipose‐derived stem cells (hADSCs) are further incorporated to form bioactive composite bioinks of Hydrogel‐DCM‐Exos and Hydrogel‐DBM‐Exos. b) Functional scaffolds are 3D‐printed and evaluated for chondrogenesis and osteogenesis in vitro. c) 3D‐printed bilayer microenvironment‐specific biomimetic scaffolds (Bi‐Hydrogel‐Exos) promote physiological regeneration of critical‐sized OC defect in a rat model via sustained release of bioactive exosomes from the double‐network hydrogel system.

Herein, we report the development of a novel spatial hydrogel scaffold comprising a 3D, bioprinted exosome‐laden bilayer that provides a tissue‐specific microenvironment with a double network. Cross‐linking with methacrylated gelatin (GelMA) results in the formation of a rigid and brittle first network, where Schiff's base bonds can establish a soft and ductile second network. We investigated the bioactivity and chondrogenic and osteogenic properties of the scaffold in vitro, alongside the regeneration of cartilage and subchondral bone in a rat model of critical‐sized OC defects (**Figure** **1**).

## 2 Results

### 2.1 Characterization of DCM, DBM, and Gelatin and Hyaluronic Acid Derivatives

**Figure 2:**
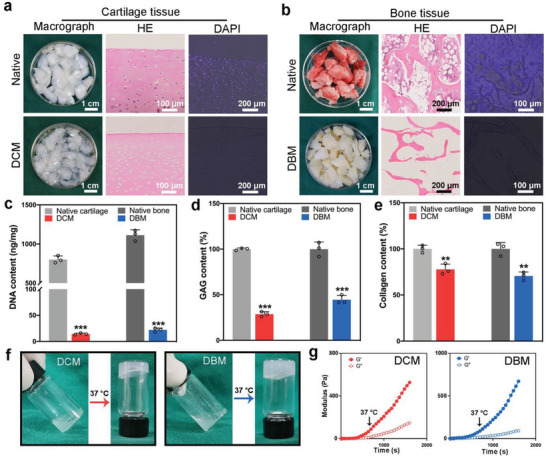
Preparation and evaluation of the dECMs. a,b) Photographs, hematoxylin‐eosin (H&E) and DAPI staining by of native cartilage and bone tissues and DCM and DBM. c–e) Changes in the levels of DANN (c), glycosaminoglycan (GAG) (d), and collagen content (e) before and after decellularization of cartilage and bone tissues. Data are presented as mean values ± SD, *n* = 3 independent biological replicates. Statistical significance was determined by two‐tailed t test. *P<0.05, **P<0.01, ***P<0.001. f), Photographs before and after gelation of the DCM and DBM pre‐gel solution at 37 °C. g) Rheological tests indicate thermosensitive sol–gel transition of DCM and DBM pre‐gel solution.

The dECM is composed of diverse macromolecules that determine its tissue‐specific biochemical properties.^[^ ^40^ ^]^ Previous results have revealed distinct protein profiles between cartilage tissues and associated growth plates.^[^ ^21^ ^]^ In this study, porcine knee cartilage and cancellous bone were both used to prepare tissue‐specific microenvironment‐biomimetic dECMs for DCM and DBM, respectively. The decellularization process involved previously described physical‐, enzymatic‐, and chemical‐treatment steps with modifications.^[^ ^41^, ^42^ ^]^ Resulting macrographs showed that the DCM was smoother and more hyaline than native cartilage tissue (**Figure** **2**a). Additionally, after decellularization, the red marrow in the bone tissue disappeared, whilst the DBM showed an elastic texture (Figure 2b). Furthermore, hematoxylin and eosin (H&E) and 4′,6′‐diamino‐2‐fenil‐indol (DAPI) staining of representative sections indicated that the DCM and DBM were successfully cleared of cells, cell debris, and nuclei (Figure 2a,b). DNA‐qualification assays also revealed decellularization efficiencies of 98.6% and 98.0% and with only 14.2 ± 1.7 and 22.0 ± 3.6 ng of DNA/mg tissue remaining in the DCM and DBM samples (Figure 2c), respectively. This met the objective of <50 ng DNA/mg tissue dry weight.^[^ ^43^ ^]^ Dimethylmethylene blue assays demonstrated that ≈30% and 45% of glycosaminoglycans (GAGs) were retained in the DCM and DBM samples, respectively (Figure 2d). Meanwhile, hydroxyproline assays indicated that > 70% of collagen was retained after decellularization (Figure 2e). The DCM and DBM samples were then digested and neutralized to form a viscous dECM solution. When warmed to a physiological temperature of 37 °C, both exhibited thermosensitive sol–gel transitions (Figure 2f), which was further supported by the results of rheological tests (Figure 2g).

Although dECMs (e.g., DCM and DBM) provide beneficial queues and a microenvironment for tissue‐specific regeneration applications without immunogenic materials, their mechanical properties limit their utility for constructing a multilayer scaffold. Pure dECM degrades quickly in vivo whilst lacking the slow‐release function of bioactive drug‐delivery vehicles. Thus, we used three types of biomaterials, namely GelMA, hyaluronic acid‐graft‐dopamine (HA‐DA), and oxidized HA (OHA) with aldehyde groups, to overcome the shortcomings of dECMs.

GelMA provides mechanical backbone support upon photoirradiation with ultraviolet (UV) light. The new proton nuclear magnetic resonance (^1^H NMR) peaks were observed at 5.3 and 5.5 parts per million (ppm), which the proton in the alkenyl group, whilst a new signal at 1.8 ppm was assigned to methyl of the introduced methacrylate group (Figure S1a, Supporting Information). The degree of methacrylation of GelMA was 28.1%. The successfully synthesized GelMA showed a decreased signal from lysine methylene (at 2.9 ppm), when compared with that of gelatin.^[^ ^44^ ^]^ Additionally, dopamine was chemically grafted to HA using 1‐(3‐dimethylaminopropyl)−3‐ethylcarbodiimide hydrochloride (EDC) and N‐hydroxysuccinimide (NHS) to synthesize HA‐DA. Mussel‐inspired or catechol‐containing hydrogel systems often exhibit good wet adhesiveness, adhesion property, and good antioxidant ability.^[^ ^45^, ^46^ ^]^ Fortunately, dopamine within HA‐DA can also simultaneously undergo self‐polymerization to generate a hydrophilic polydopamine (PDA), which reinforces the dispersity of nanoparticles (e.g., exosomes), enhances the integration of nanointerface between collagens and nanoparticles, and prolongs their release as gatekeepers.^[^ ^47^, ^48^ ^]^ Our ^1^H NMR results showed new peaks at 6.7 and 2.76 ppm, which could be assigned to the representing catechol ring and the –CH_2_ group close to the catechol ring, respectively (Figure S1b, Supporting Information). The DA content of HA‐DA was detected as 11.7%, from the absorbance at 280 nm using a standard curve of DA by UV–vis spectrometry (Figure S2, Supporting Information). To synthesize OHA, the aldehyde groups were conjugated to HA via a chemical reaction with sodium periodate to synthesize OHA. The new peaks at 4.9 and 5.0 ppm were then detected through ^1^H NMR analysis (Figure S1b, Supporting Information), which corresponded to hemiacetalic protons from the aldehyde group and adjacent hydroxyls.^[^ ^49^ ^]^ The oxidation degree of OHA was calculated to be 24.6% as quantified by measuring the number of aldehydes using hydrochloride potentiometric titration method.

### 2.2 Physical Characteristics of 3D‐Printed Microenvironment‐Specific Biomimetic Hydrogel Scaffolds

We used GelMA, HA‐DA, and OHA to form a double‐network bioink system referred to here as the Hydrogel system, which consisted of 9% GelMA, 2% OHA, and 2% HA‐DA. Because the two primary inks are mixed to build a composite ink, the concentration of the primary ink is twice that of the final ink. 12%, 18%, and 24% of GelMA primary ink were prepared in the pre‐experiment to obtain 6%, 9%, and 12% of the final concentration ink. However, 24% GelMA ink is too sticky even at 37 °C, and cannot be mixed with other ingredients. Considering the feasibility of preparation, we chose GelMA with 18% concentration as the primary ink. Based on relevant literature reports, 2% to 3% concentration of OHA and HA‐DA can play the role of dynamic crosslinking of Schiff base and promote cell adhesion and dispersion of nanoparticles.^[^ ^46^, ^50^ ^]^

The addition of DCM or DBM into the Hydrogel system formed microenvironment‐specific biomimetic Hydrogel‐DCM or Hydrogel‐DBM bioinks, respectively. To optimize the dECM concentrations, we detected the rheological properties of different bioinks with 1%, 2%, and 3% dECM (Figure S3, Supporting Information). We evaluated the gelation kinetics of the bioinks at varying temperatures starting from 10 °C to 40 °C (Figure S3a,b, Supporting Information). The gel–sol transition temperature was around 35, 32, 31, and 30 °C in Hydrogel, Hydrogel‐dECM (3%), Hydrogel‐dECM (2%), and Hydrogel‐dECM (1%), respectively. Meanwhile, the data clearly demonstrated that both storage (G′) and loss (G′′) modulus values and viscosity increased with an increment of the dECM. All bioinks showed decreased viscosity in the measured shear rate range (Figure S3c, Supporting Information), indicating the shear thinning flow behavior which can be smoothly squeezed by 3D printing. The photocurable propriety was significantly improved with the incorporation of dECM, although there was no difference between 2% and 3% groups (Figure S3d, Supporting Information), demonstrating a higher mechanical characteristic of the Hydrogel‐dECM (2%) and Hydrogel‐dECM (3%) bioinks. Based on the above properties, we decided to choose 2% dECM for subsequent experiments. Subsequently, Hydrogel, Hydrogel‐DCM, and Hydrogel‐DBM scaffolds, which consisted of 9% GelMA, 2% HA‐DA, 2% OHA, and 2% DCM or DBM, were all fabricated using an extrusion‐based 3D printing method and were crosslinked using UV light (Figure S4, Supporting Information). We used these concentrations because they provided a modest viscosity whilst helping to avoid unstable preparations of high‐viscosity bioinks and excessive pressure during the extrusion‐based printing process. The presence of GelMA rendered the printing process highly controllable and stable. During plotting, the needle diameter, layer thickness, and fiber space were all held constant at 320 µm, which was suitable for cell attachment, proliferation, and differentiation.^[^ ^51^ ^]^

The chemical compositions of the different hydrogels were subsequently assessed using Fourier transform infrared (FTIR) spectroscopy. A stretching vibration was observed at 1735 cm^−1^ for the aldehyde group (–CHO) in OHA, whereas this signal was absent with the Hydrogel, Hydrogel‐DCM, and Hydrogel‐DBM scaffolds (Figure S5, Supporting Information), indicating that the dynamic bond was successfully formed through a Schiff's base reaction. GelMA cross‐linking resulted in the formation of a rigid and brittle first network, whereas the Schiff's base bond served as the soft and ductile second network. Scanning electron microscopy (SEM) images (Figure S6, Supporting Information) showed that Hydrogel‐DCM and Hydrogel‐DBM scaffolds contained denser and more porous structures in filaments compared to Hydrogel scaffolds. This demonstrated that DCM and DBM micelles improved the morphological characteristics of the 3D‐printed scaffolds. To examine the effect of DCM and DBM on the properties of the scaffolds, we measured variation in their degrees of swelling and degradation at different times. Importantly, the dECM micelles improved the swelling ratio and time required to reach a swelling equilibrium for the Hydrogel‐DCM and Hydrogel‐DBM scaffolds, when compared with those of the Hydrogel scaffolds (Figure S7a, Supporting Information). The degradation behaviors of the Hydrogel, Hydrogel‐DCM, and Hydrogel‐DBM scaffolds were evaluated by detecting the percentage of residual mass. As shown in Figure S7b (Supporting Information), the degradation rate decreased significantly with the addition of DCM or DBM.

### 2.3 Evaluating the Biocompatibility of Scaffolds, Exosomal Characteristics, and Release Profile of Exosomes in Vitro

**Figure 3:**
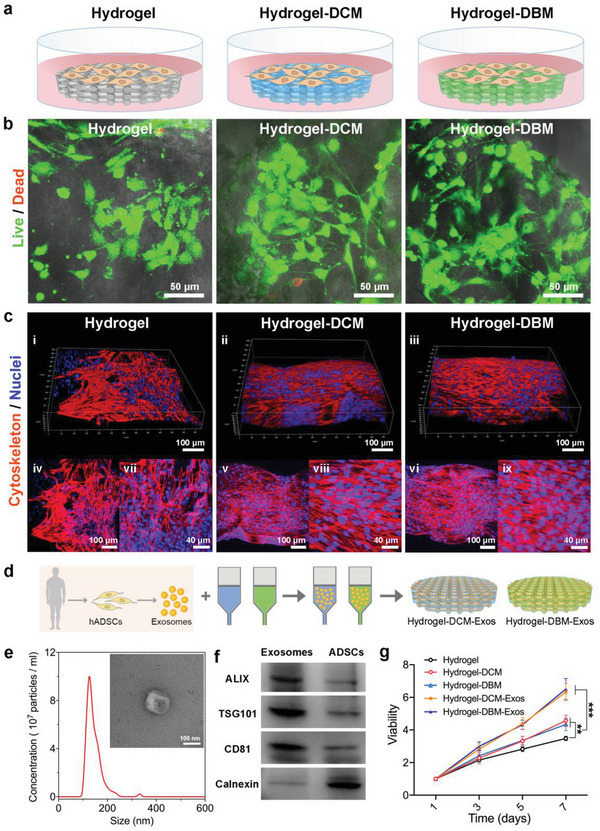
Biocompatibility of different scaffolds. a) rBMSCs are seeded in different scaffolds to assess the biocompatibility of scaffolds. b) CLSM images of Live/Dead assay indicates the Hydrogel, Hydrogel‐DCM, and Hydrogel‐DBM scaffolds are friendly on rBMSCs after being seeded for 24 h. c) Cytoskeleton stained by rhodamine–phalloidin shows better attachment and outspread ability of rBMSCs in Hydrogel‐DCM and Hydrogel‐DBM scaffolds than those in Hydrogel scaffold after 72 h growth. i–iii) 3D images of rBMSCs in the alinement of different scaffolds; iv–vi) stacked images of cytofilaments in Hydrogel, Hydrogel‐DCM, and Hydrogel‐DBM scaffolds; vii–ix) enlarged view from different scaffolds. Scale bars are indicated in the images. d) Exosomes from human adipose‐derived stem cells (hADSCs) were isolated and encapsulated into the Hydrogel‐DCM and Hydrogel‐DBM bioinks to print bioactive Hydrogel‐DCM‐Exos and Hydrogel‐DBM‐Exos scaffolds. e) Nanoparticle tracking analysis and TEM scanning (the inserted image, scale bar = 100 nm) for exosomes isolated from hADSCs. f) Western blotting analysis for exosome specific protein markers. g) Cell viability assay of rBMSCs in exosome‐encapsulated scaffolds indicates the best proliferative capacities after incubated for 7 days *n* = 5 independent biological replicates. Statistical significance was determined by two‐way ANOVA test. *P<0.05, **P<0.01, ***P<0.001.

Rat bone marrow MSCs (rBMSCs) were seeded in the printed scaffolds and cultured in growth medium to assess their biocompatibility (**Figure** **3**a). After being incubated for 24 h, live/dead assays were detected via confocal laser scanning microscopy (CLSM), which showed that the rBMSCs grew well on each type of scaffold, with no obvious cell death observed in any group (Figure 3b). Considering that the cytoskeleton in the matrix dramatically affects cellular responses, we examined the relationship between rBMSC morphology and 3D scaffolds. The rBMSCs were stained after incubation for 72 h with rhodamine–phalloidin and DAPI to visualize actin filaments and nuclei, respectively. Subsequently, CLSM images of rBMSCs on the Hydrogel‐DCM and Hydrogel‐DBM scaffolds displayed an orderly spindle‐shaped morphology with extended typical pseudopodia, whereas cells on the Hydrogel scaffold exhibited irregularly aggregated actin filaments (Figure 3c). Overall, these results revealed that DCM and DBM dECMs promoted cell attachment and spreading, whilst also helping to maintain the morphology of cytofilaments. Taken together, these results revealed that the Hydrogel, Hydrogel‐DCM, and Hydrogel‐DBM scaffolds were all non‐toxic to the rBMSCs.

In view of the recognized capability of exosomes to facilitate chondrogenic and osteogenic differentiation of MSCs, the vesicles derived from human adipose‐derived stem cells (hADSCs) were encapsulated into the Hydrogel‐DCM and Hydrogel‐DBM bioinks in order to print bioactive Hydrogel‐DCM‐Exos and Hydrogel‐DBM‐Exos scaffolds (Figure 3d). hADSC‐derived exosomes were used here since they can efficiently induce cartilage and bone tissue formation through signal‐transduction pathways mediated by their bioactive cargo proteins, as reported in our previous study.^[^ ^36^ ^]^ Human knee infrapatellar fat pads were emulsified to isolate hADSCs, as described previously.^[^ ^52^ ^]^ We identified hADSCs based on their multi‐lineage differentiation potential and the positive expression of CD29, CD73, CD90, and CD105 (Figures S8,S9, Supporting Information); thus, the hADSCs were compliant with the criteria of the International Federation for Adipose Therapeutics and Science and the International Society for Cellular Therapy (ISCT).^[^ ^53^ ^]^ Exosomes purified from the culture medium of hADSCs showed a typical saucer‐shaped vesicular morphology with a bilayer‐membrane structure when viewed under a transmission electron microscope (TEM, Figure 3e), which was similar to previous findings.^[^ ^54^ ^]^ Nanoparticle tracking analysis revealed that the hADSC‐derived exosomes had an average diameter of 140.3 nm (Figure 3e), which was in accordance with our TEM results alongside previous data recorded regarding exosome‐size distributions.^[^ ^28^ ^]^ Furthermore, Western blotting analysis demonstrated that the vesicles positively expressed the exosome‐specific marker proteins ALIX, TSG101, and CD81, whereas they did not express the endoplasmic reticulum protein Calnexin (Figure 3f).

The exosomes were stained with the PKH67 dye to visualize their distributions in the scaffolds. CLSM images subsequently revealed that the encapsulated exosomes were distributed evenly in the filaments of the printed scaffolds (Figure S10a,b, Supporting Information). The cumulative‐release efficiency of exosomes from the printed Hydrogel‐DCM‐Exos and Hydrogel‐DBM‐Exos scaffolds were both ≈80% after 24 days (Figure S10c, Supporting Information). Next, we evaluated rBMSC proliferation in the scaffolds (Figure 3g) to assess the bioactivity of the released exosomes. The cells in each group exhibited elevated viability over time. Specifically, the rBMSCs in the Hydrogel‐DCM and Hydrogel‐DBM groups showed markedly higher proliferation than those in the Hydrogel group. Moreover, the groups with encapsulated exosomes showed significantly higher proliferative capabilities than the other groups, demonstrating that the released exosomes maintained their bioactivity and increased cell proliferation. Chemotaxis is defined as migration of cells in response to specific stimuli or biological signals. Furthermore, we conducted a Transwell assay to evaluate the effect of released exosomes from scaffolds on cell migration behavior. The data showed that the most superior migrated cell counts in Hydrogel‐DCM‐Exos and Hydrogel‐DBM‐Exos groups (Figure S11, Supporting Information), indicating the released exosomes bioactively promote cell chemotaxis. Overall, our results showed that all hydrogel scaffolds exhibited good biocompatibility and non‐cytotoxicity with rBMSCs. Furthermore, the 3D microenvironment‐specific biomimetic scaffolds facilitated cell extension and proliferation. Additionally, the combination of bioactive exosome release enhanced the ability to modulate rBMSC proliferation and migration.

### 2.4 Chondrogenic‐ and Osteogenic‐Differentiation Bioactivities of rBMSCs on Printed Scaffolds in Vitro

**Figure 4:**
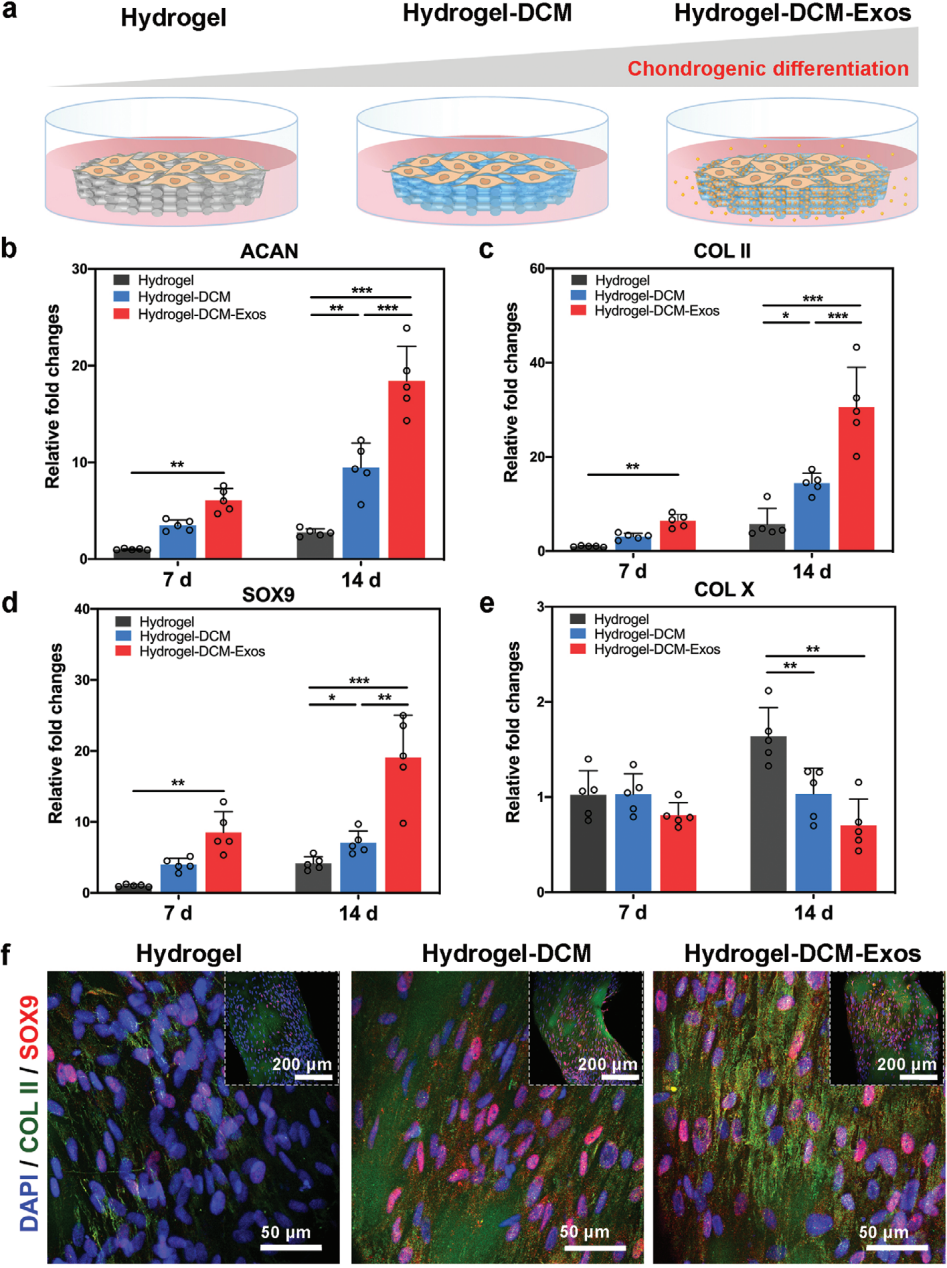
Gene and protein analysis for chondrogenic differentiation from rBMSCs grown in different scaffolds. a) rBMSCs were seeded in the Hydrogel, Hydrogel‐DCM, and Hydrogel‐DCM‐Exos scaffolds and incubated in chondrogenic medium. The expression of cartilage‐associated b) aggrecan (ACAN), c) collagen type II (COL II), d) SRY‐box transcription factor 9 (SOX9), and e) COL X gene in rBMSCs detected by real‐time quantitative polymerase chain reaction (RT‐qPCR) after incubated for 7 or 14 days, respectively. *n* = 5 in each group, three independent experiments. Statistical significance was determined by non‐parametric test. *P<0.05, **P<0.01, ***P<0.001. f) Immunofluorescence (IF) images show the expression of COL II and SOX9 proteins at 14 d in chondrogenic medium. The inset image represents a low‐magnification panorama containing the hydrogel alinement. *n* = 3 independent biological replicates.

To elucidate the effect of the 3D printed scaffolds on chondrogenic differentiation (**Figure** **4**a), real‐time quantitative polymerase chain reaction (RT‐qPCR) analysis was performed to detect hyaline cartilage‐specific gene‐expression levels (Figure 4b–e). The expression levels of cartilage‐specific genes, including aggrecan (ACAN), collagen type II (COL II), and SRY‐box transcription factor 9 (SOX9), were all significantly upregulated in rBMSCs cultured on Hydrogel‐DCM and Hydrogel‐DCM‐Exos scaffolds after induction for 7 and 14 days, relative to those in the Hydrogel scaffold, demonstrating that the presence of both exosomes and DCM synergistically enhanced chondrogenesis in vitro. In addition, the selected genes were expressed at significantly higher levels in rBMSCs on the Hydrogel‐DCM scaffold than in rBMSCs incubated on the Hydrogel scaffold. Expression of the hypertrophy‐related gene (COL X) was distinctly lower after both 7 and 14 days in the Hydrogel‐DCM and Hydrogel‐DCM‐Exos groups than in the Hydrogel group (Figure 4e), implying that both DCM and exosomes in the scaffolds have good potential for suppressing the hypertrophic differentiation of rBMSCs. Immunofluorescence (IF) staining of the COL II and SOX9 proteins was analyzed by CLSM after 14 days of growth in chondrogenic medium. Similar to the RT‐qPCR results, the COL II and SOX9 proteins were expressed most abundantly in rBMSCs cultured on Hydrogel‐DCM‐Exos scaffolds, followed by rBMSCs cultured on Hydrogel‐DCM scaffolds, and finally by rBMSCs cultured on Hydrogel scaffolds (Figure 4f; Figure S12, Supporting Information). These findings ultimately demonstrated that Hydrogel‐DCM‐Exos scaffolds exhibited an augmented capability to promote the chondrogenic differentiation of rBMSCs in vitro.

**Figure 5:**
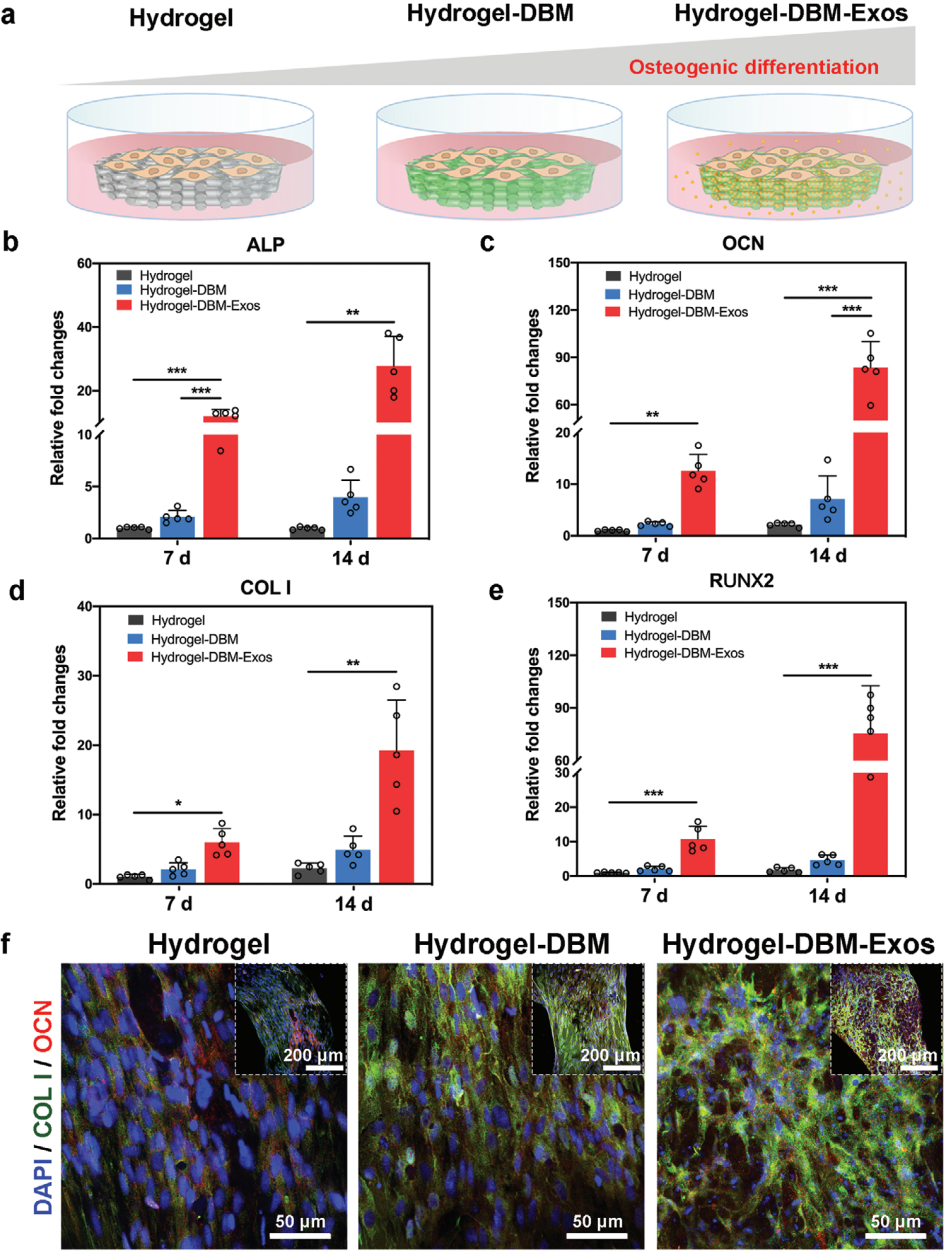
The expression of bone‐related genes and proteins in rBMSCs grown in different scaffolds. a) rBMSCs were seeded in the Hydrogel, Hydrogel‐DBM, and Hydrogel‐DBM‐Exos scaffolds and incubated in osteogenic medium. b–e) RT‐qPCR analysis of bone‐associated gene (b) alkaline phosphatase biomineralization associated (ALP), (c) osteocalcin (OCN), (d) COL I, and (e) RUNX family transcription factor 2 (RUNX2) after a 7 or 14 days osteogenic incubation, respectively. *n* = 5 in each group, three independent experiments. Statistical significance was determined by non‐parametric test. *P<0.05, **P<0.01, ***P<0.001. f) IF images show the expression of COL I and OCN protein after incubated for 14 days. The inset image represents a low‐magnification view containing the hydrogel alinement. n = 3 independent biological replicates.

To assess the effect of scaffolds on promoting osteogenic differentiation (**Figure** **5**a), we next investigated the expression of bone‐related genes, including alkaline phosphatase biomineralization associated (ALP), osteocalcin (OCN), COL I, and RUNX family transcription factor 2 (RUNX2), after 7 and 14 days of osteogenic induction (Figure 5b–e). ALP plays a key role in the early mineralization of osteogenic differentiation.^[^ ^8^ ^]^ Compared to the Hydrogel scaffold, ALP expression of rBMSCs on both Hydrogel‐DBM and Hydrogel‐DBM‐Exos scaffolds was markedly upregulated, with an increases of 12 and 27 fold being observed in the Hydrogel‐DBM‐Exos group after incubation for 7 and 14 d, respectively (Figure 5b). After incubation for 14 d, the expression of OCN and COL I in the Hydrogel‐DBM‐Exos group was considerably higher than that in other groups (Figure 5c,d). RUNX2 is the main transcription factor involved in osteoblast differentiation. Significantly higher upregulated gene expression levels of RUNX2 were also observed in the Hydrogel‐DBM‐Exos group compared to the Hydrogel and Hydrogel‐DBM groups on days 7 and 14 (Figure 5e). After osteogenic incubation for 14 days, IF imaging and quantitative analysis of COL I and OCN proteins (Figure 5f; Figure S13, Supporting Information) were conducted. The results subsequently demonstrated that the scaffolds with encapsulated exosomes had the highest efficiency of osteogenesis in vitro, which was consistent with previous RT‐qPCR data.

Taken together, these data confirmed that the dECMs acted as environment‐specific cues to effectively facilitate MSC differentiation into their corresponding lineage and that the sustained release of exosomes from Hydrogel‐DCM‐Exos and Hydrogel‐DBM‐Exos scaffolds could promote the specific differentiation of stem cells in their biomimetic zones.

### 2.5 In Vivo Cartilage and Subchondral Bone Repair Efficacy of the Printed Scaffolds

**Figure 6:**
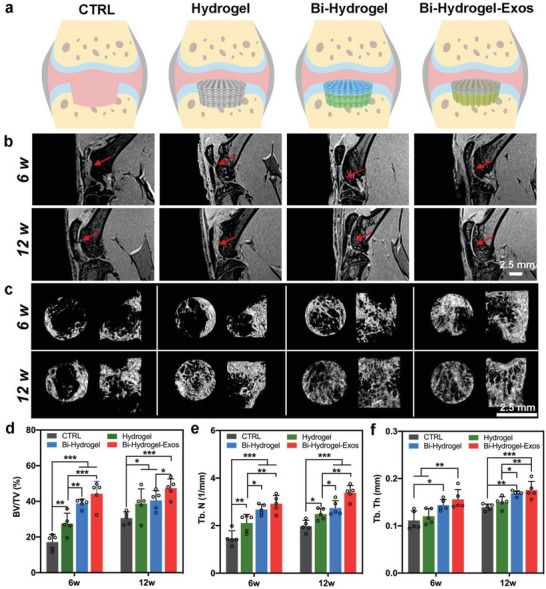
Imageological analysis of the defects at weeks 8 and 12 after surgery. a) An overview illustrating 3D‐printed different scaffolds implanted in rat knee osteochondral defects. b) Representative MRI images showing chondrogenesis in the defect at 6 and 12 weeks postoperatively. The arrows show the defect sites. c) Representative 3D reconstructed micro‐CT images reveal the regeneration of subchondral bone at defect sites in blank control (CTRL), Hydrogel, Bi‐Hydrogel, and Bi‐Hydrogel‐exos scaffold implanted animals. d–f) Quantitative analysis of (d) bone volume/total volume (BV/TV), (e) trabecular number (Tb.N), and (f) trabecular thickness (Tb.Th) for regenerated bone tissues in the defects. *n* = 5 individual rats, *P<0.05, **P<0.01, ***P<0.001.

Subsequently, we printed Hydrogel‐DBM and Hydrogel‐DCM as the bone phase and cartilage phase, respectively, to form a spatial microenvironment‐biomimetic Bi‐Hydrogel scaffold. Similarly, the exosome‐containing Hydrogel‐DBM‐Exos and Hydrogel‐DCM‐Exos were successively printed layer‐by‐layer to form the bioactive Bi‐Hydrogel‐Exos scaffold (**Figure** **6**a). We performed an in vivo experiment to evaluate the effect of different scaffolds on cartilage and subchondral bone regeneration using a rat model with critical‐sized OC defects. To evaluate new cartilage formation, magnetic resonance imaging (MRI) was performed 6 and 12 weeks after surgery (Figure 6b). Specifically, at 6 weeks post‐operation, the defects in the blank control (CTRL) group were poorly filled with irregular newly formed tissue, whereas the sites in the Hydrogel and Bi‐Hydrogel groups were filled with discontinuously repaired tissue. However, the injured areas in the Bi‐Hydrogel‐Exos group were almost completely filled with newly formed cartilage tissue, although not up to the joint surface.

At 12 weeks, the injuries in the CTRL group were irregularly filled, with a discontinuous rough surface below the normal joint surface. Although the generated tissue was continuous in the Hydrogel group, its signal was not consistent with that of the surrounding tissue, suggesting that hyaline cartilage was not fully formed here. In the Bi‐Hydrogel and Bi‐Hydrogel‐Exos groups, the repaired tissues showed a similar MRI signal to the surrounding normal joint, indicating that hyaline cartilage was formed at the injured sites. The defects were fully filled with a smooth surface and vague boundary in the Bi‐Hydrogel‐Exos group, whereas a distinct margin was observed in the Bi‐Hydrogel group, suggesting that the regenerative capacity was enhanced when the exosomes were encapsulated in Bi‐Hydrogel‐Exos scaffolds.

Micro‐computed tomography (micro‐CT) was performed at 6 and 12 weeks after surgery to evaluate subchondral bone regeneration, with Figure 6c displaying representative 3D‐reconstructed images. At 6 weeks, the CTRL and Hydrogel groups still had large defect areas with relatively little new bone generation, whereas the Bi‐Hydrogel and Bi‐Hydrogel‐Exos groups showed obvious bone tissue coverage in the defects. At 12 weeks, a large area remained unrepaired in the CTRL group, whilst bone tissue formation in the defects of the Hydrogel group increased significantly but not by as much as in the Bi‐Hydrogel and Bi‐Hydrogel‐Exos groups.

Next, a quantitative analysis of the reconstructed images was performed. The bone volume/total volume (BV/TV) and trabecular number (Tb.N) values of the scaffold‐implanted groups were found to be significantly higher than those of the CTRL group at both 6 and 12 weeks after surgery (Figure 6d,e). The Bi‐Hydrogel and Bi‐Hydrogel‐Exos groups showed significantly higher BV/TV and Tb.N values than the Hydrogel group at 6 weeks, although only the Bi‐Hydrogel‐Exos group maintained this trend when compared with the Hydrogel group at 12 weeks, suggesting that dECM played was critical for early‐stage bone regeneration. The Tb.N values of the Bi‐Hydrogel‐Exos group were significantly higher than those in the Bi‐Hydrogel group (Figure 6e), indicating that the early osteogenic effect of the released bioactive exosomes supported bone regeneration. In addition, both the Bi‐Hydrogel and Bi‐Hydrogel‐Exos groups displayed significantly higher trabecular thickness (Tb.Th) values than the other groups at 12 weeks (Figure 6f).

The gross appearance revealed more cartilage‐like tissue filling in the defects in the Bi‐Hydrogel and Bi‐Hydrogel‐Exos groups, whereas obvious tissue indentation remained at the defect sites in the CTRL group without scaffold implantation and Hydrogel groups at 6 weeks post‐operation (Figure S14, Supporting Information). At 12 weeks after surgery, the regenerated tissue in the Bi‐Hydrogel‐Exos group showed a color and texture similar to that of normal cartilage, with a glossier and well‐integrated morphology, indicating that hyaline cartilage‐like tissue successfully formed. The Bi‐Hydrogel group also showed a well‐integrated morphology but was not as smooth as the surrounding normal cartilage tissue. However, several small defects were still observed in the Hydrogel group, whilst large indentations without cartilage formation were observed in the CTRL group at 12 weeks post‐operation (Figure S14, Supporting Information).

**Figure 7:**
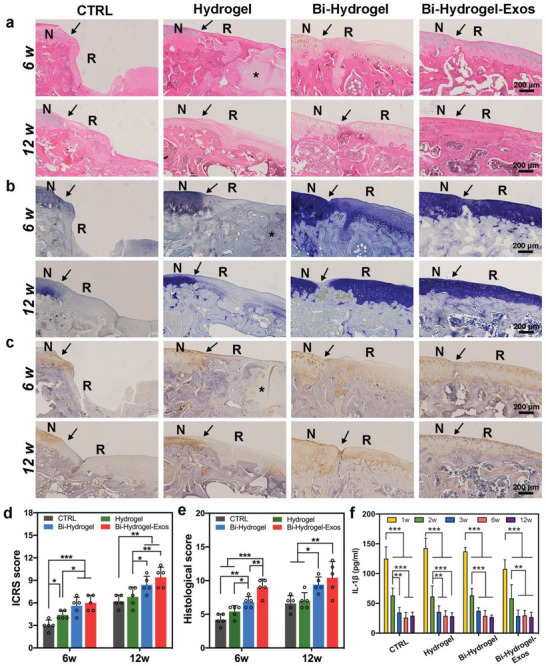
Histological assessment of repaired tissues after 6 and 12 weeks post‐operation in different groups. Representative images of a) H&E, b) toluidine blue staining, and c) immunohistological staining for type II collagen. The black arrows show the edges of the defects. The asterisks indicate the residual scaffold. N: normal cartilage; R: repaired cartilage. d,e) The International Cartilage Repair Score (ICRS) (d) and the histological score (e) for repaired tissues in each group after 6 and 12 weeks post‐operatively. f) Quantitative analysis of interleukin‐1β (1L‐1β) in the serum of experimental animals at 1, 2, 3, 6, and 12 weeks after surgery. *n* = 5 individual rats, *P<0.05, **P<0.01, ***P<0.001.

H&E staining was performed to assess the general structure of regenerative tissues in each group (**Figure** **7**a). After 6 weeks post‐implantation, the CTRL group showed the most prominent tissue indentation at the defect sites among all groups, where few regenerated tissues were formed, with the boundary between the newly formed tissues and the surrounding normal tissues being distinct. Defects implanted with Hydrogel scaffolds exhibited fibrous tissues and started subchondral bone regeneration, with residual scaffolds at the detection sites (asterisk, Figure 7a). The Bi‐Hydrogel group showed more newly regenerated subchondral bone tissue, cartilage tissue regeneration, and fibrous tissue on the surface, with slight disruptions. The implanted Bi‐Hydrogel‐Exos scaffold revealed better hyaline cartilage regeneration, with a smooth cartilage surface and no obvious boundaries at the defect sites. At week 12, the margin between the neo‐tissue and native cartilage remained obvious in the CTRL and Hydrogel groups, whereas little boundary was observed between the Bi‐Hydrogel and the well‐integrated neo‐tissue and adjacent cartilage in the Bi‐Hydrogel‐Exos group. Meanwhile, the Bi‐Hydrogel‐Exos scaffold group exhibited the most continuous subchondral bone formation.

Toluidine blue (TB) staining (Figure 7b) and immunohistological staining of COL II (Figure 7c) were both performed to assess the cellular matrix, structural integrity, and tissue regeneration. Weak staining signals were observed with the regenerated neo‐tissues and surrounding native cartilage tissues in the CTRL and Hydrogel groups, indicating that there was little newly formed hyaline cartilage and that the adjacent normal cartilage underwent degradation. Meanwhile, the lacuna structure was barely observable in the CTRL and Hydrogel groups. However, the structures of the newly formed subchondral bone and cartilage were not similar to those of natural tissue, whilst a clear boundary was present between the newly formed tissues and the surrounding normal tissues. Nonetheless, abundant round cells were embedded in the newly formed lacunae, indicating that mature hyaline cartilage was regenerated in the Bi‐Hydrogel and Bi‐Hydrogel‐Exos groups, although a minor margin appeared between the neo‐cartilage and native cartilage in the Bi‐Hydrogel group at 12 weeks post‐implantation. The International Cartilage Repair Score (ICRS) and the histological score both showed the same trend (Figure 7d,e), with the Bi‐Hydrogel and Bi‐Hydrogel‐Exos groups demonstrating markedly higher scores at 6 and 12 weeks post‐implantation than the Hydrogel and CTRL groups. Distinct higher ICRS and histological scores were observed in the Bi‐Hydrogel‐Exos group than in the Bi‐Hydrogel group at 12‐weeks post‐implantation, although a significant difference in their histological scores was observed at 6 weeks post‐implantation, indicating that the released exosomes mainly promoted tissue regeneration at an early stage. Levels of the inflammatory factor interleukin 1β (IL‐1β) decreased sharply in whole blood serum samples after 2 weeks (Figure 7f), suggesting that IL‐1β production was mainly caused by the operation.^[^ ^55^ ^]^ In addition. IL‐1β level at 1 week post‐operation was significantly down‐regulated in Bi‐Hydrogel‐Exos group than that in the Bi‐Hydrogel and Hydrogel groups (Figure S15, Supporting Information), suggesting the released bioactive exosomes can play a benefit effect on reducing inflammatory reaction.

**Figure 8:**
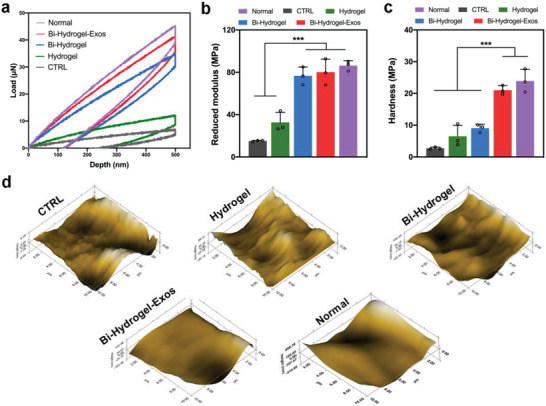
Biomechanical properties of repaired cartilage at 12 weeks post‐operation. a) Typical load‐displacement curves of normal cartilage and the repaired tissues in each group. b,c) Reduced modulus and hardness of the regenerated tissues and normal cartilage. d), Microscopic geomorphology of the repaired sites. *n* = 3 individual rats, ***P<0.001.

At 12 weeks post‐surgery, the biomechanical properties of the reparative cartilage were assessed by nanoindentation test (Figure S16, Supporting Information). According to the load‐displacement curves (**Figure** **8**a), reduced modulus and hardness were calculated. The CTRL and Hydrogel groups displayed lower reduced modulus than the Bi‐Hydrogel and Bi‐Hydrogel‐Exos groups which were similar to normal cartilage (Figure 8b). However, only the Bi‐Hydrogel‐Exos group showed a comparable hardness property than normal cartilage (Figure 8c), indicating exosomes can significantly improve the biomechanical restoration for joint injury. In addition, using micro‐scanning, the articular surface repairs in the Bi‐Hydrogel‐Exos scaffold group was the most integrated and smooth, followed by the Bi‐Hydrogel group, and the worst (more scraggier and rougher) in the CTRL and Hydrogel groups (Figure 8d). These data indicate that the Bi‐Hydrogel‐Exos scaffold facilitates better biomechanical properties in the repaired articular hyaline cartilage tissue.

In addition, the cytotoxicity of the scaffolds was evaluated in vivo. H&E staining demonstrated that the anatomy of the main organs in each group remained normal at 6 and 12 weeks post‐operation (Figure S17, Supporting Information). Routine blood analysis revealed no obvious systemic toxicity in any group (Figure S18, Supporting Information). Therefore, the scaffolds were safe for use in rats.

## 3 Discussion

Here, we demonstrated that a 3D‐printed tissue‐specific microenvironment biomimetic scaffold could efficiently enhance chondrogenic and osteogenic stem cell differentiation in vitro as well as promoting articular cartilage and subchondral bone regeneration in a rat model of critical‐sized OC defects. With the sustained release of hADSC‐derived exosomes, the biomimetic scaffold presented here could be used to deliver cargo molecules beneficial for cartilage and bone growth and thus served as a novel cell‐free recipe for stem cell therapy. These results highlight the potential benefits of microenvironmental biomimetic bilayer scaffolds administered in combination with bioactive extracellular vesicles to regenerate cartilage and bone tissues.

Although technically pluripotent, MSCs rarely repair damaged tissue in vivo through direct differentiation and engraftment due to certain limitations, including the reduced capacity of these cells for self‐renewal, proliferation, and differentiation in donor sites.^[^ ^56^ ^]^ The true benefit of MSCs in tissue repair appears to be attributable to their paracrine secretion of cellular factors, particularly exosomes, that modulate the microenvironment.^[^ ^57^ ^]^ Recently, interest has arisen in the use of MSC‐derived exosomes as a biological tool for cartilage and bone repair. Previously, we and others have demonstrated that exosomes mediated osteochondral differentiation of stem cells and that their bioactive cargo molecules influenced stem cell behavior and lineage determination.^[^ ^35^, ^36^, ^39^, ^58^, ^59^, ^60^ ^]^ Multiple injections of exosomes are effective, but increase the risk of infection whilst posing the disadvantage of faster diffusion in the joint cavity, making them unsuitable for clinical translation. Moreover, recent evidence has indicated that the kinetics of exosome delivery can impact tissue regeneration at the molecular, cellular, and tissue levels.^[^ ^61^ ^]^ Therefore, we previously explored the encapsulation of exosomes into biomaterials and showed that the slow release of these bioactive vesicles facilitated the regeneration of damaged tissues.^[^ ^29^, ^62^ ^]^ Previous data has shown that dECM is a good natural biomaterial that can promote cartilage regeneration by stimulating endogenous stem cell recruitment.^[^ ^18^, ^19^, ^63^ ^]^ However, in terms of complex tissue restoration, recent evidence has indicated that lineage‐specific dECM is more advantageous.^[^ ^21^, ^64^ ^]^ In addition, ECM from different regions of tissues has different properties, whereas dECM inherits this feature. For example, the dECM of the inner meniscus enhanced the fibrocartilaginous differentiation of MSCs, whereas the dECM of the outer meniscus promoted a more fibroblastic phenotype.^[^ ^20^ ^]^ One recent report showed that modifying the freeze‐drying kinetics and direction of heat transfer when freezing bilayer dECM scaffolds improved their repair outcomes with OC defects.^[^ ^65^ ^]^ Our results demonstrated the feasibility of using a 3D‐printing strategy with region‐specific dECM of cartilage and bone tissues as bioink ingredients to fabricate dECM scaffolds that could be used to induce microenvironment‐specific differentiation or a tissue‐specific lineage.

More importantly, for the first time, here we combined tissue‐specific dECM with exosomes to fabricate a spatially biomimetic OC scaffold using 3D‐printing technology. The addition of DCM or DBM to 3D‐printed scaffolds promoted chondrogenesis or osteogenesis seeded with rBMSCs. Owing to the GelMA and dynamic Schiff's base bonds in the double‐network hydrogel, the exosomes achieved a sustained release from the printed scaffolds, achieving much longer release kinetics than surface modification and simple encapsulation methods.^[^ ^39^, ^66^ ^]^ Furthermore, the released exosomes efficiently synergized with 3D dECM‐based microenvironment‐specific scaffolds to promote chondrogenic or osteogenic rBMSC differentiation in vitro by enhancing key transcription factors and inhibiting cell hypertrophy. This conclusion was further supported by a previous observation that exosomes isolated from osteoblasts and adipocytes augmented dECM‐mediated MSC differentiation.^[^ ^67^ ^]^ Previous results showed that 3D printing and photocrosslinking scaffolds with exosome‐containing bioinks helped to maintain the release of exosomes to promote cell migration,^[^ ^68^ ^]^ which supports our finding that exosomes released from 3D‐printed scaffolds functionally regulated cellular processes, including differentiation. Previous findings have also demonstrated that exosomes released from scaffolds promoted cartilage or bone defect repair, whilst fewer studies have been conducted to investigate articular cartilage and subchondral bone tissues simultaneously. The results of this study provide both in vitro and in situ evidence that the sustained release of MSC‐derived exosomes greatly promotes OC injury healing. It has been also previously reported that MSC‐derived exosomes can inhibit inflammatory responses. We found a significant decrease of IL‐1β level at 1 week post‐surgery in the Bi‐Hydrogel‐Exos group, demonstrating the released exosomes can improve inflammatory microenvironment.

The current work provides proof of concept that 3D printing of a microenvironment biomimetic scaffold based on tissue‐specific dECM, in association with MSC‐derived exosomes, could be used to facilitate simultaneous cartilage and bone restoration in OC defects. Our findings also prompt investigation into the suitability of printing dECM bioinks for scaffolds and provide credible evidence for the crossing the milestone of translating 3D bioprinting into clinical settings.^[^ ^69^ ^]^ However, several limitations and challenges still need to be overcome. First, there is still a need to accurately illustrate which molecules within the released exosomes play important functional roles. As a cocktail of various bioactive cargo molecules, the exosomes in our tissue‐specific microenvironment bionic scaffolds may have promoted chondrogenesis and osteogenesis through different mechanisms. Second, we found no difference in the histological scores between the Bi‐Hydrogel and Bi‐Hydrogel‐Exos groups at 12 weeks after implantation. There are several possible explanations for these this findings. The in vitro‐release profile showed that ≈80% of exosomes were released after 24 days, whilst there is a possibility exists that the bioactive vesicles mainly carried out synergistic effects during the early stage. Natural exosomes cannot specifically target MSCs in vivo; thus, in situ cartilage and bone generation are not as efficient as in vitro. Another limitation of this study was that we did not use a large‐animal model. Future studies should i) focus on the molecular mechanisms whereby the released exosomes induced chondrogenesis and osteogenesis, ii) optimize the release profile of exosomes and the degradation regimen of scaffolds to achieve the best cartilage and bone healing in vivo, and iii) test engineered exosomes with an MSC‐targeted delivery function or specific bioactive molecules to further enhance their efficacy in large animals.

## 4 Conclusion

In summary, here we propose a new concept for preparing tissue‐specific microenvironment bionic hydrogel scaffolds that can achieve excellent cartilage and subchondral bone tissue regeneration in large OC defects. This strategy provides an insight into the clinical translation of 3D printing and exosome‐reinforced cell‐free MSC therapeutics as off‐the‐shelf implants for joint regeneration. This study also suggests a biomimetic strategy and innovation for designing structured and functionalized 3D bioinspired scaffolds for the targeted regeneration of complex tissues.

## 5 Experimental Section

Experimental methods can be found in the Supporting Information. All animal experimental protocols were approved by Peking University Biomedical Ethics Committee (No. LA2020021), and the methods in this work were carried out in accordance with the Guidelines for the Care and Use of Laboratory Animals. For human adipose‐derived mesenchymal stem cells (hADSCs) isolation, the study was approved by the Medical Ethics Committee of Peking University Third Hospital (No. M2020311 and 2013003).

## Author Contributions

Q.L. conceived the research, performed in vitro and in vivo experiments, prepared the figures, and wrote the manuscript. H.Y., F.Z., and C.C. performed some in vitro experiments and analyzed the data. T.W., and Y.F. involved in some critical work. Y.A., and X.H. supervised the research and reviewed the manuscript.

## Conflict of Interest

The authors declare no conflict of interest.

## Supporting information

Supporting Information

## Data Availability

The data that support the findings of this study are available from the corresponding author upon reasonable request.
