# Supplementary material for: 3D Printing of Microenvironment‐Specific Bioinspired and Exosome‐Reinforced Hydrogel Scaffolds for Efficient Cartilage and Subchondral Bone Regeneration
Source: Adv Sci (Weinh). 2023 Jul 9;10(26):2303650. doi: 10.1002/advs.202303650 (PMC10502685; doi:10.1002/advs.202303650)
Supplement: Supplementary file 1 — Supporting Information [file ADVS-10-2303650-s001.pdf]

ADVANCED SCIENCE
Open Access

Supporting Information

for *Adv. Sci.*, DOI 10.1002/advs.202303650

3D Printing of Microenvironment-Specific Bioinspired and Exosome-Reinforced Hydrogel Scaffolds for Efficient Cartilage and Subchondral Bone Regeneration

Qi Li, Huilei Yu, Fengyuan Zhao, Chenxi Cao, Tong Wu, Yifei Fan, Yingfang Ao and Xiaoqing Hu**

Supporting Information

3D Printing of Microenvironment-Specific Bioinspired and Exosome-Reinforced Hydrogel Scaffolds for Efficient Cartilage and Subchondral Bone Regeneration

Qi Li, Huilei Yu, Fengyuan Zhao, Chenxi Cao, Tong Wu, Yifei Fan, Yingfang Ao, Xiaoqing Hu**

Dr. Q. Li, Dr. H. Yu, Dr. F, Zhao, Dr. C. Cao, Dr.T. Wu, Y. Fan, Prof. Y. Ao, Prof. X. Hu

Department of Sports Medicine, Institute of Sports Medicine of Peking University, Beijing Key Laboratory of Sports Injuries, Peking University Third Hospital, Beijing 100191, China

Email: aoyingfang@163.com (Y. Ao); huxiaoqingbd01@sina.com (X. Hu)

Dr. Q. Li

Center of Foot and Ankle Surgery, Beijing Tongren Hospital, Capital Medical University, Beijing 100730, China

Materials and Methods

The following chemicals were purchased from Sigma-Aldrich (St. Louis, MO, USA): gelatin from porcine skin (type A, Cat. G2500), methacrylic anhydride (Cat. 276685), dopamine hydrochloride (Cat. H8502), Trizma-HCl (Cat. T3253), Trizma base (Cat. T6791), SDS (Cat. V900859), DNase I (Cat. DN25), RNase A (Cat. 10109169001), EDTA (Cat. E6758), Triton X-100 (Cat. V900502), pepsin (Cat. P7125), papain (Cat. P3125), DMMB (Cat. 341088), and sulfated glycosaminoglycan polysaccharide (Cat. C4384). N-hydroxysuccinimide (NHS; Cat. H109330), EDC (Cat. E106172), sodium periodate (Cat. S104093), and ethylene glycol (Cat. E103322) were purchased from Aladdin (Shanghai, China). Sodium hyaluronate (Cat. S24592) was purchased from Yuanye Bio-Technology (Shanghai, China). Hydroxylamine hydrochloride (Cat. H811237) and methyl orange (Cat. M812777) were purchased from Macklin (Shanghai, China). 0.25% trypsin-EDTA (Cat. 25200072) and Minimum Essential Medium Alpha (MEM-α) were purchased from Gibco (Grand Island, NY, USA). Dulbecco's modified phosphate buffered saline (DPBS) with no calcium or magnesium (Cat. PYG14190) was obtained from BOSTER (Wuhan, China). All reagents were used without further purification. Rat interleukin-1β (IL-1β; Cat. RLB00) ELISA kit were purchased from R&D Systems (Minneapolis, MN, USA).

Cell Isolation and Culture

Rat bone marrow mesenchymal stem cells (rBMSCs) from Sprague-Dawley (SD) rats (80-100g) were isolated and identified as described previously.[1] Briefly, bone marrow from the distal femur and tibia were aspirated by DPBS through an 18-gauge needle.

After centrifugation, cell pellets were obtained by centrifugation and resuspended in MEM-α containing 10% fetal bovine serum (FBS; Gibco) and 1% penicillin/streptomycin. Cells were maintained at 37°C in a humidified incubator containing 5% CO_2. The medium was not replaced until 5 days later, and then changed every three days until the cells were grown to 90% confluency. The cells at passages 3-4 were used for the following experiments.

For human adipose-derived mesenchymal stem cells (hADSCs) isolation, the study was approved by the Medical Ethics Committee of Peking University Third Hospital (No. M2020311 and 2013003). Written informed consents were signed. The adipose tissue is collected from the discarded subpatellar fat pad of a patient undergoing the arthroscopic anterior cruciate ligament reconstruction surgery. The hADSCs were isolated as previously described.[2] Cells were grown in MEM-α supplemented with 10% FBS and 1% penicillin/streptomycin. The medium was changed every three days until the cells reached 90% confluency. Cells were cryopreserved in liquid nitrogen for further experiments.

Flow Cytometry

hADSCs at passage 2 were digested and centrifuged, washed twice with DPBS, and then incubated with primary antibody for one hour at room temperature in the dark. Cell suspension was incubated with primary antibodies against CD29 (eBioscience, 11-0299-42), CD34 (Santa Cruz Biotechnology), CD45 (eBioscience, MA5-28383), CD73 (eBioscience, 11-0739-42), CD90 (BD Pharmingen, 555595), CD105 (Exbio, 1P-298) or HLA-DR (eBioscience, 11-9952-42). After incubated with CD34 and CD73

antibodies, FITC conjugated anti-mouse IgG secondary antibody (BioLegend, 405305, San Diego, CA, USA) was incubated for one hour. Stained cells were then analyzed using a FACSVerse flow cytometer (BD Biosciences).

Synthesis of Gelatin Methacryloyl (GelMA)

GelMA was synthesized according to a previously reported protocol.[3] Briefly, 6 g of gelatin was dissolved in 60 mL of DPBS at 50 °C. Once fully dissolved, 300 μL methacrylic anhydride was added dropwise to the solution and allowed to homogenize. After 1 h of vigorous stir, 200 mL of pre-heated DPBS at 50 °C was added to the solution to inactivate the reaction. Then, the solution was dialyzed against deionized (DI) water via a dialysis cassette with molecular weight cut-off (MWCO) of 12-14 kDa for 3 days. DI water was changed twice daily. The lyophilized GelMA was kept dry and stored at −20 °C until use. The degree of methacrylation of GelMA was 28.1%, determined by integration of ^{1}H NMR spectra. The ratio of the integrated intensities of lysine methylene at 2.8-2.95 ppm in the spectra of GelMA and gelatin was calculated.[4]

Synthesis of Oxidative Hyaluronic Acid (OHA)

OHA was synthesized according to an already reported method.[5] Briefly, HA (1 g) was dissolved in DPBS (100 mL) and stirred evenly at room temperature (RT). 550 mg of sodium periodate ($NaIO_4$) was mixed with 5 mL of DPBS under mild stirring at RT. Then, the $NaIO_4$ solution was added dropwise to the HA solution and stirred for 2 h at RT in the dark. 1 ml of ethylene glycol was then added to cease the unreacted periodate for 1 h. After exhaustive dialysis against DI water for 3 days, OHA was lyophilized by

freeze-drying and stored at −20 °C before use. The oxidation degree of OHA was 24.6%, defined by hydroxylamine hydrochloride potentiometric titration method.[6]

Synthesis of Dopamine-Conjugated Hyaluronic Acid (HA-DA)

HA-DA was synthesized as previously described.[7] Briefly, 1 g of HA was dissolved into 100 mL of DPBS and agitated until completely dissolved. 575 mg of EDC and 375 mg of NHS were slowly added to the solution and agitated for 20 min. Then, 569 mg of dopamine hydrochloride was added to the mixture. The pH value of the solution was monitored and adjusted to keep the value between 5 and 6 for 3 h. Then, the mixture was allowed to react overnight at RT. Following dialyzed against DI water for 3 days under acidic conditions and lyophilized, the obtained HA-DA was stored −20 °C until use. Aqueous solutions were prepared and the ultraviolet spectra at 240 nm to 400 nm were measured by UV-vis spectrometry. The degree of DA substitution in HA-DA was calculated as 11.7% by measuring the absorbance at 280 nm using a standard curve of DA.

Decellularization of Cartilage and Bone

Porcine knees from a 6-month-old pig was collected from a nearby slaughter house and used with approval from the supplier. Cartilage was decellularized according to a previously reported protocol with modification.[8] Briefly, articular cartilage was sliced into pieces of about 1 mm in thickness using a surgical knife blade and 6 cycles of freezing in liquid nitrogen and thawing at 37 °C were conducted. Decellularized cartilage matrix (DCM) was prepared by a series of washes under vigorous agitation (250 RPM): 1) 0.25% trypsin-EDTA solution for 6 h × 4 at 37 °C; 2) 10 mM Trizma-

HCl, 50 U mL^{-1} DNase, and 1 U mL^{-1} RNase A in DPBS at 37 °C for 4 h; 3) 10 mM Trizma-HCl in DPBS for 24 h; 4) 0.5% SDS in DPBS for 24 h; 5) 1% Triton X-100 solution for 24 h; 6) DPBS for 12 h × 6 to remove all the detergent. The obtained DCM pieces were then snap frozen, lyophilized and stored at −20 °C until required.

Bone tissue was decellularized by following the protocol published elsewhere with modification.[9] In brief, trabecular bone from the knee joint was cut into small cubes using a bone saw and washed with a high-pressure faucet to remove bone marrow. Decellularized bone matrix (DBM) was prepared by a series of procedures at 250 RPM: 1) 0.5 N HCl for 24 h at room temperature for demineralization; 2) a 1:1 mixture of chloroform and methanol for 6 h, methanol for 6 h, and DPBS for 2 h to remove the lipid; 3) 0.25% trypsin-EDTA solution for 6 h × 4 at 37 °C; 4) 0.1% EDTA, 10 mM Trizma base, 50 U mL^{-1} DNase, and 1 U mL^{-1} RNase A in DPBS for 4 h at 37 °C; 5) 0.1% EDTA, 10 mM Trizma base in DPBS for 24 h; 6) 0.5% SDS, 10 mM Trizma base in DPBS for 24 h; 7) DPBS for 12 h × 6 to remove all the detergent. The obtained DBM pieces were then snap frozen, lyophilized and stored at −20 °C until required.

Biochemical Characterization of dECMs

To evaluate the degree of decellularization, samples of native or decellularized cartilage and bone tissues were separately pulverized into micro-particles using a cryogenic ball mill (Retsch CryoMill, Germany), and then filtered through a 40-μm cell strainer to obtain the fine powder. 10 mg of samples were then digested in 1 mL of prepared papain extraction solution (125 μg/mL papain enzyme, 5 mM Na_2-EDTA, 0.1 M sodium acetate, and 5 mM cysteine-HCl at pH 6.2) at 60 °C for 24 h. Aliquots of the sample

digestion were incubated in dark at 37 °C for 1 h with 200 μL of Hoechst 33258 working solution (2 μg/mL). The DNA content was normalized with a standard curve made using DNA from calf thymusand (Sigma-Aldrich, Cat. D8899). The fluorescence intensity was then measured using a Varioskan Flash multimode microplate reader (Thermo Fisher Scientific, USA) at an excitation wavelength of 360 nm and an emission wavelength of 460 nm. Histological sections were analyzed with H&E staining and DAPI staining.

For GAGs quantification, the DMMB assay was performed as previously described. Briefly, samples of native or decellularized cartilage and bone tissues (10 mg) were digested at 60°C for 24 h in 1 ml papain extraction solution (125 μg/ml papain enzyme, 5 mM Na_2-EDTA, 0.1 M sodium acetate, and 5 mM cysteine-HCl at pH 6.2). Lysates (20 μL) were mixed with 200 μL of DMMB working solution for 30 min at RT in the dark. The standard curve was made using sulfated glycosaminoglycan polysaccharide from shark cartilage (Sigma-Aldrich, Cat. C4384). The absorbance was then measured at 525 nm with a microplate reader.

The total collagen content was estimated using a hydroxyproline assay kit (A030, Nanjing Jiancheng Bioengineering Institute, China) according to the instructions. Samples in papain extraction solution was used and reacted with the working solution. The absorbance of final supernatant was detected at wavelength of 560 nm with a microplate reader.

Preparation of dECM solution

DCM and DBM pieces were separately pulverized into micro-particles using a cryogenic ball mill (Retsch CryoMill), and then filtered through a 40-μm cell strainer to obtain the fine powder. Required amount of dECM (DCM or DBM) powder was weighed and digested in a solution of 0.1 N HCl solution supplemented with 10 mg of pepsin for 100 mg dECM for 72 h to prepare 4% (wt/v) DCM or DBM bioinks. Then, the pH was adjusted to 7.4 with dropwise addition of 10 N NaOH solution on an ice bath to prevent gelation. Rheological characteristic of DCM and DBM bioinks was detected using a HAAK MARS rheometer (Thermo Scientific).

Preparation of Bioinks

Preparation of Hydrogel bio-ink: 0.9 g of GelMA was weighed and dissolved in 5 mL of DI water at 37 °C, then 0.2 g of OHA was added and stirred till complete dissolution. 0.2 g of HA-DA was weighed and dissolved in 5 mL of DI water at RT, then 0.04 g photoinitiator Irgacure 2959 was added and stirred thoroughly in the dark. The two solutions were then mixed and homogenized to obtain Hydrogel bio-ink.

Preparation of Hydrogel-DCM and Hydrogel-DBM bioinks: 0.9g of GelMA was weighed and dissolved in 5 mL of DI water at 37 °C, then 0.2 g of OHA was added and stirred till complete dissolution. 0.2 g of HA-DA and 0.04 g photoinitiator Irgacure 2959 were added into 5 mL of newly prepared 4% (wt/v) DCM and DBM bioinks (pH = 7.4) and stirred in ice bath. The two solutions were then mixed and homogenized to obtain Hydrogel-DCM and Hydrogel-DBM bioinks, respectively.

Preparation of Hydrogel-DCM-Exos and Hydrogel-DBM-Exos bioinks: 500 μg freshly purified exosomes were mixed with 5 mL of Hydrogel-DCM and Hydrogel-

DCM bioinks and homogenized to obtain Hydrogel-DCM-Exos and Hydrogel-DBM-Exos bioinks, respectively.

Rheological Test of Bioinks

Different rheological characteristics of hydrogels were carried out on a rheometer (HAKKE MASE Ш, Germany). The C35 1° /Ti cone rotor was used to perform the experiments with a truncation gap distance of 52 μm. Temperature sweep analysis was conducted for characterizing the thermal response of the bioinks. It was conducted with temperature rate of +2.4 °C min^{-1} in a range of 10 °C–40 °C at 1 Hz. The viscosity of the bioinks was measured through shear rate sweeps, which were conducted over the frequency range from 0.01 to 1000 s^{-1}. UV-triggered oscillatory time sweep tests were performed on an external UV module with a glass plate rotor (20 mm diameter, 1 mm gap). All samples were measured at 10% strain and 1 Hz for 300 s and UV light was emitted from 60 s.[10]

3D Printing of Scaffolds

The scaffolds were fabricated by sequential strand deposition method using a 3D-Bioplotter (EnvisionTEC, Germany). The structural design was programmed by SolidWorks software (Autodesk, USA). The bioinks were filled into the cartridge and kept at 22 °C for 1 h before printing. Whereafter, the strands were plotted onto an acrylic sheet at a plotting speed of 15 mm/s under a pressure of about 350 kPa for Hydrogel printing and 250 kPa for Hydrogel-DCM, Hydrogel-DBM, Hydrogel-DCM-Exos, and Hydrogel-DBM-Exos printing. During plotting, the needle diameter, layer thickness, and layer width were all kept constant at 320 μm. The printing chamber and

stationary platform were kept at a constant temperature of 22 °C and 10 °C, respectively. Each layer adhered to the underlying layer perpendicularly to form a 0°/90° strut structure. For in vitro experiments, a total of six layers of scaffolds were printed. For in vivo study, a total of nine layers of Hydrogel, Bi-Hydrogel and Bio-Hydrogel-Exos scaffolds were printed under the previously described printing parameters. Specifically, six layers of Hydrogel-DBM or Hydrogel-DBM-Exos were printed as subchondral bone layer, followed by three layers of Hydrogel-DCM or Hydrogel-DCM-Exos to form cartilage layer. Polymerization and sterilization were further carried out on ice bath for 20 min in an CL-1000 Ultraviolet crosslinking oven (UVP, USA).

Nuclear magnetic resonance (NMR) analysis

Required amount of gelatin, GelMA, HA, OHA, and HA-DA were weighed and dissolved in D_2O to prepare corresponding hydrogel of 1% (wt/v) gelatin, 1% (wt/v) GelMA, 0.8% (wt/v) HA, 0.8% (wt/v) OHA, and 0.8% (wt/v) HA-DA. Samples were carefully transferred into NMR tubes without bubbles. The ^1H NMR spectra were acquired on a Bruker Avance III 600 MHz spectrometer. All solutions were prepared in D_2O. The ^1H NMR spectrum of D_2O at 4.74 ppm was used as reference.

Fourier Transform Infrared (FTIR) Spectrometry

Freeze-dried samples and printed scaffolds were crushed to powder and prepared by the KBr tablet method. Then, a FTIR spectrometry (Thermo Scientific, USA) was used to derecte from 4000 to 400 cm^{-1} for confirming the successful synthesis of Hydrogel, Hydrogel-DCM, and Hydrogel-DBM.

Scanning Electron Microscopy (SEM)

Morphological characteristics of the printed Hydrogel, Hydrogel-DCM, and Hydrogel-DBM scaffolds were observed using SEM. Freeze-dried scaffolds were sputter-coated with a 5-nm layer of gold in a high-vacuum gold sputter coater, and were then examined using a scanning electron microscope (JSM-7900F, JEOL, Tokyo, Japan).

Swelling Behavior Test

The freshly printed Hydrogel, Hydrogel-DCM, and Hydrogel-DBM scaffolds were weighted to record their initial wet weights (W_i) and then separately immersed in 1ml of PBS at 37 °C. At indicated time intervals, the scaffolds were removed from the medium, and the excess solvent on the surface was gently blotted before recoding swollen wet weights (W_t). The swelling ratio of the scaffolds was calculated by: $(W_t - W_i) / W_i \times 100\%$. All experiments were performed in triplicate.

In Vitro Degradation Test

The degradation behaviors of the printed scaffolds were detected in PBS with collagenase I (1 μg/mL) and collagenase II (1 μg/mL) to simulate the environments of joints. After the initial dry weight of the freeze-dried 3D printed scaffolds with 1cm length, 1 cm width, and 1.8 mm thickness were recorded, the samples were incubated in 1ml of enzymatic solution at 37 °C and 30 rpm per min. The medium was replaced every 3 days. At predetermined time intervals, the degraded samples were removed, washed two times, and lyophilized. The initial and final weight of the freeze-dried sample were measured as W_i and W_t, respectively. The degradation ratio of the scaffolds was calculated by: $(W_i - W_t) / W_i \times 100\%$. All experiments were performed in triplicate.

Cytocompatibility of Scaffolds

The cytotoxicity of scaffolds was detected by employing a direct contact test between scaffolds and rBMSCs. Prior to the cell culture, the scaffolds were immersed in growth medium with 10% exosome-free FBS overnight. rBMSCs were seeded with an initial density of 1×10^6 on each scaffold in 12-well cell culture plates containing 1 mL of MEM-α medium supplemented with 10% exosome-free FBS and 1% penicillin/streptomycin. After incubation for 24 h, cell viability in the scaffolds culture was assessed with a Live/Dead Viability Kit assay (Invitrogen, Carlsbad, CA, USA). Images were captured via a confocal laser scanning microscope (CLSM; SP8, Leica, Germany).

Isolation and Identification of Exosomes

hADSCs at passage 4 were grown in Falcon 875 cm^2 multi-flasks (Corning, Oneonta, NY, USA) until approximately 80% confluency. After rinsing twice with PBS, cells were refreshed with MEM-α containing 5% exosome-free FBS for 48 h. The conditioned medium (CM) was collected and exosomes were isolated from the cell supernatant by differential centrifugation. Briefly, the CM was centrifuged at 300 × g and 2000 × g for 10 min to remove cells and cell debris, respectively. After centrifuged at 10,000 × g for 30 min to discard large vesicles, the resulting supernatant was filtered with a 0.22μm filter (Merck Millipore). Then, exosomes were isolated by ultracentrifugation at 100,000 × g and 4 °C for 2 h using an Optima l-XP ultracentrifuge. Further, the obtained pellets were rinsed with cold PBS and ultracentrifuged again. Size distribution within exosome samples was analyzed by nanoparticle tracking analysis

(NTA).[1] The morphology of the extracted exosomes was observed using transmission electron microscopy (TEM; JEM-1400, JEOL, Tokyo, Japan). The CD81, ALIX, and TSG101 molecules which frequently located on the surface of exosomes and endoplasmic reticulum protein Calnexin were analyzed using Western blotting. Primary antibodies were as follows: anti-CD81 (1: 1000; Abcam, ab109201, Cambridge, UK), anti-ALIX (1:1000; Proteintech, 12422-1-AP, USA), anti-TSG101 (1: 1000; Proteintech, 14497-1-AP), and anti-Calnexin (1: 1000; Abcam, ab133615). The bands were detected by chemiluminescent HRP reagent (Merck Millipore) and the signals were visualized by a ChemiDoc XRS+ Imaging System (Bio-Rad, Hercules, CA, USA).

Release Profile of Exosomes

The exosome release profile was tested by a micro BCA protein assay kit (Thermo Scientific, USA).[11,12] Briefly, the above prepared Hydrogel-DCM, Hydrogel-DCM-Exos, Hydrogel-DBM, or Hydrogel-DBM-Exos scaffold was placed in the upper chamber of the Transwell insert (8 μm, Corning, USA) placed in a 12-well plate, while PBS was added in the lower chamber. Then 15 μL PBS was collected and replaced by 15 μL fresh PBS every three days. Three scaffolds were performed in each group and the measurement performed in triplicate. The Hydrogel-DCM or Hydrogel-DBM scaffold was also detected as a substrate control to correct the effect of protein degradation in the system. The content of released exosomes was detected, and the exosomes released profile was calculated and expressed as the released percentage along with time.

Labeling of Exosomes

Exosomes were labeled with the membrane dye using a PKH67 Green Fluorescent dye (Sigma-Aldrich) according to the manufacturer's instructions. Briefly, 0.5 mL exosomes in PBS (1 μg/μL) were suspended in 0.5mL diluent reagent, then mixed with 1 mL PKH67 dye working solution (0.4% in diluent reagent). After incubated for 5min, 2 mL of 1% BSA for each reaction was added to stop reaction. Then, samples were rinsed with cold PBS and centrifuged at 100,000 × g and 4 °C for 2 h. The labeled exosomes were further used to print Hydrogel-DCM-Exos scaffolds and visualized by the SP8 CLSM (Leica, Germany).

Cell Counting Kit-8 (CCK8) Assay

Cell proliferation on the scaffolds was determined using a CCK8 (Dojindo Laboratories, Kumamoto, Japan) assay at days 1, 3, 5, and 7. Briefly, rBMSCs were seeded with an initial density of 1×10^6 on each scaffold in 24-well cell culture plates containing 1 mL of MEM-α medium supplemented with 10% exosome-free FBS and 1% penicillin/streptomycin. The cell-seeded scaffolds were cultured in a humidified incubator containing 5% CO_2 with the culture medium changed every 3 days. At the desired time points, the culture medium was replaced with 1mL of MEM-α containing 10% CCK-8 reagents and incubated at 37 °C for 2 h. 100 μL of medium was transferred in a 96-well cell culture plate to detect the absorbance at 450 nm in a microplate reader.

Cell migration

Cell migration behavior was evaluated by the Transwell assay. Briefly, 2×10^5 rBMSCs were seeded in the upper chamber of the Transwell insert and placed in a 24-well plate,

while different scaffolds were immersed in the lower chamber with MEM-α medium (supplemented with 1% exosome-depleted FBS). After incubation at 37°C for 12 or 24 h, cells on the underside of the filter were fixed with 4% paraformaldehyde, stained with 0.1% crystal violet, and photographed under a microscope. Cell counting was calculated using ImageJ software (National Institutes of Health, USA).

Cell Differentiation

rBMSCs were seeded with an initial density of 1×10^6 on each scaffold in 24-well cell culture plates containing 1 mL of MEM-α medium supplemented with 10% exosome-free FBS and 1% penicillin/streptomycin. After a 3-d cultivation in the incubator, cell-seeded scaffolds were rinsed twice with PBS and incubated with differentiation. For chondrogenic induction, ITS (insulin-transferrin-selenium), dexamethasone, L-ascorbic acid, L-proline, sodium pyruvate, and transforming growth factor β3 were supplemented in the basal medium. For osteogenic induction, dexamethasone, L-ascorbic acid, β-glycerophosphate, glutamine, penicillin-streptomycin, and 10% exosome-depleted FBS were supplemented in the basal medium. The differentiation medium was replaced every 3 days.

Quantitative Real-Time Reverse-Transcriptase Polymerase Chain Reaction (qRT-PCR)

After incubation for 7 and 14 d, total RNA was extracted in TRIzol reagent (Invitrogen) and isolated using a Direct-zol RNA MiniPrep kit (Zymo Research, Irvine, CA, USA). Then, 0.5 μg of total RNA was reverse transcribed to cDNA using the FastKing RT Kit with gDNase (Tiangen Biotech, Beijing, China). Then, qRT-PCR was performed using

an ABI QuantStudio 3 Real-Time PCR System (Applied Biosystems, CA, USA) with the PowerUp SYBR Green Master Mix (Applied Biosystems). The house-keeping gene 18S ribosomal RNA (Rn18s) was used to normalize the relative mRNA expression level of each gene using the the $2^{-\Delta\Delta Ct}$ method. Primer information is listed in Table S1 (Supporting Information).

Immunofluorescence

After chondrogenic or osteogenic induction for 14 days, each scaffold was rinsed with PBS for 3 times and fixed with 4% paraformaldehyde for 30 min at RT. Scaffolds were then permeabilized with 0.1% Triton X-100 for 30 min and incubated with 10% goat serum for 1 h to block nonspecific binding sites. Then, the scaffolds were incubated against primary antibodies overnight at 4 °C. In detail, the cocktailed primary antibodies included COL II (1:200; Abcam, ab34712) and SOX9 (1:200; Novus, NBP2-52943, USA) were adopted for the chondrogenic induced Hydrogel, Hydrogel-DCM, and Hydrogel-DCM-Exos scaffolds, while the cocktail of primary antibodies was COL I (1:200; Abcam, ab34710) and OCN (1:200; Abcam, ab13418) for the osteogenic induced Hydrogel, Hydrogel-DBM, and Hydrogel-DBM-Exos scaffolds. Goat anti-rabbit IgG H&L (Alexa Fluor 488; 1:500; Abcam, ab150077) and anti-mouse IgG H&L (Alexa Fluor 568; 1:500; Abcam, ab175473) were applied for 1 h and nuclei were stained with DAPI for 15 min at RT. After rinsed with PBS, cells in scaffolds were immersed in mounting solution and observed by a CLSM. Statistical analysis was conducted by measuring the mean pixel intensity using ImageJ (NIH, USA) software.

Animal Experiments

All animal experimental protocols were approved by Peking University Biomedical Ethics Committee (No. LA2020021), and the methods in this work were carried out in accordance with the Guidelines for the Care and Use of Laboratory Animals. Male SD rats weighting 400-450 g and 12-week age were used for studies. All animals were acclimatized for 1 week before surgery and randomly divided into 4 groups, corresponding to untreated group (CTRL), printed Hydrogel scaffold, printed Bi-Hydrogel scaffold, and printed Bi-Hydrogel-Exos scaffold. A rat osteochondral defect model (2.5 mm in diameter and 3 mm in depth) was carried out to evaluate the regenerative capacities of implanted scaffolds. In detail, anesthesia was performed with inhalation of isoflurane with an animal-specific anesthesia apparatus (RWD, China). The surgery was conducted under aseptic conditions. With an incision made on the knee from the lateral side, the joint was exposed after the patella was dislocated. Then, a cylindrical defect (2.5-mm diameter, 3-mm depth) was created on the trochlear groove of the distal femur using corneal trephine. Afterwards, the printed scaffolds were implanted and the joint was closed with a suture. Penicillin was administered intramuscularly for 3 days to avoid infection. All rats were given free access to diets and sacrificed at 6 weeks or 12 weeks post-operation. For the biochemical analysis, the whole blood and serum were collected at 1, 2, 3, 6, and 12 weeks post-surgery. Routine blood parameters were detected using a hematology analyzer (Nihon Kohden, Japan). Inflammatory factor in serum was detected by the rat IL-1β and TNF-α ELISA kits followed manufacturers' instructions.

Magnetic Resonance Imaging (MRI) and Micro Computed Tomography (micro-CT) Analysis

At 6 and 12 weeks post-operation, all the animals were sacrificed. The knee samples were collected and assessed with a Siemens TIM Trio 3.0 T (T) MRI scanner (Siemens, Erlangen, Germany) using a small animal-specific coil. Then, the muscles and attached soft tissues were removed, and the distal femur was preserved and photographed. The International Cartilage Repair Society (ICRS) grading system (Table S2, Supporting Information) was used and blindly scored by 2 observers to evaluate the regenerated cartilage. After fixed in 10% neutral formalin for 48 h, the samples (n = 5 each group) were assessed using an Inveon Micro-CT system (Siemens, Germany). Scanning parameters were set at 10.3 μm resolution, 60 kV voltage, and 400 μA current. The 3D reconstruction of images and the micro-architectural parameters including relative bone volume (BV/TV), bone mineral density (BMD, mg/cm^3), trabecular number (Tb.N), and trabecular thickness (Tb.Th) were analyzed using the Inveon Research Workplace III software (Siemens).

Histological experiments

The fixed specimens were decalcified by a decalcifying solution (ZSGB-BIO, Beijing, China) for 48 h and dehydrated by a gradient alcohol series. After embedded in paraffin, histologic sections of 5-μm thickness were prepared and stained with hematoxylin and eosin (H&E) and and toluidine blue (TB). For immunohistochemical staining, the sections were immersed in 3% hydrogen peroxide in PBS to block endogenous peroxidase, and then incubated with PBS containing 10% goat serum to block

nonspecific protein bindings. Subsequently, the sections were incubated with COL II antibody (1:200; ab34712, Abcam) at 4 °C overnight. A modified Wakitani grading system (Table S3, Supporting Information) was used to score the histological repair outcomes by the same 2 observers in a blinded manner.

Nanoindentation Assessment

Biomechanical properties of regenerated tissues were evaluated using nanoindentation tests at 12 weeks (n = 3 in each group) post-operatively as described previously.[2]

Statistical Analysis

Statistical analysis data are presented as means ± standard deviation. Analyses were assessed by Student's t-tests (two groups), one-way ANOVA (homogeneity of variance, three or more groups), or non-parametric test (uneven variance) using SPSS version 22.0 (IBM, Chicago, IL, USA). *P* value < 0.05 was considered significant.

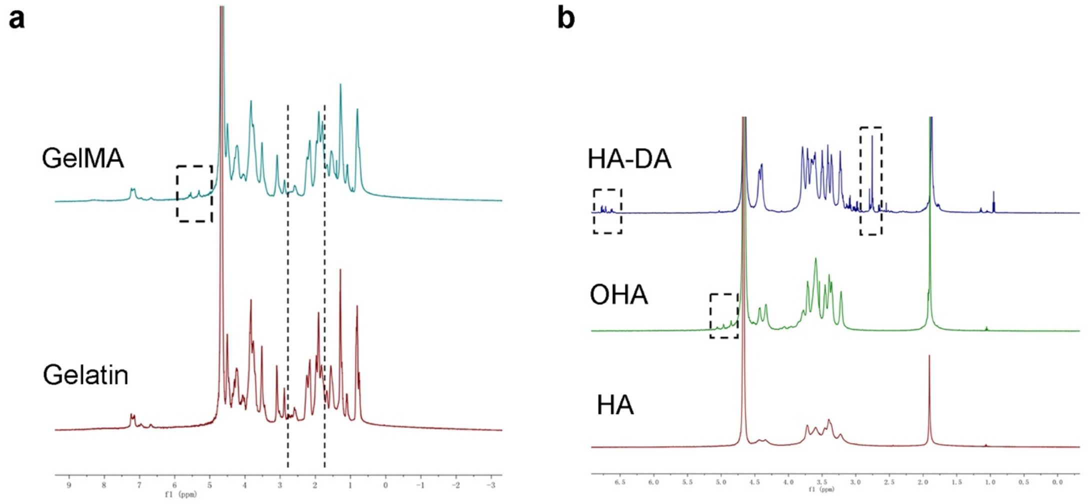

Figure S1. ^{1}H NMR analysis. **a,** ^{1}H NMR results of sgelatin and GelMA. **b**, ^{1}H NMR results of HA, OHA, and HA-DA.

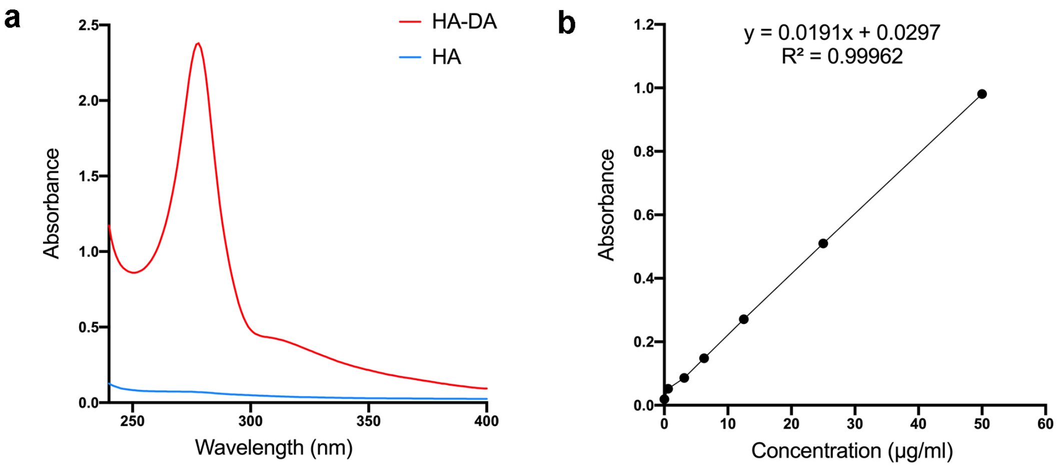

Figure S2. a, UV-Vis spectra of HA and HA-DA. **b,** Standard curve of DA measured at 280 nm.

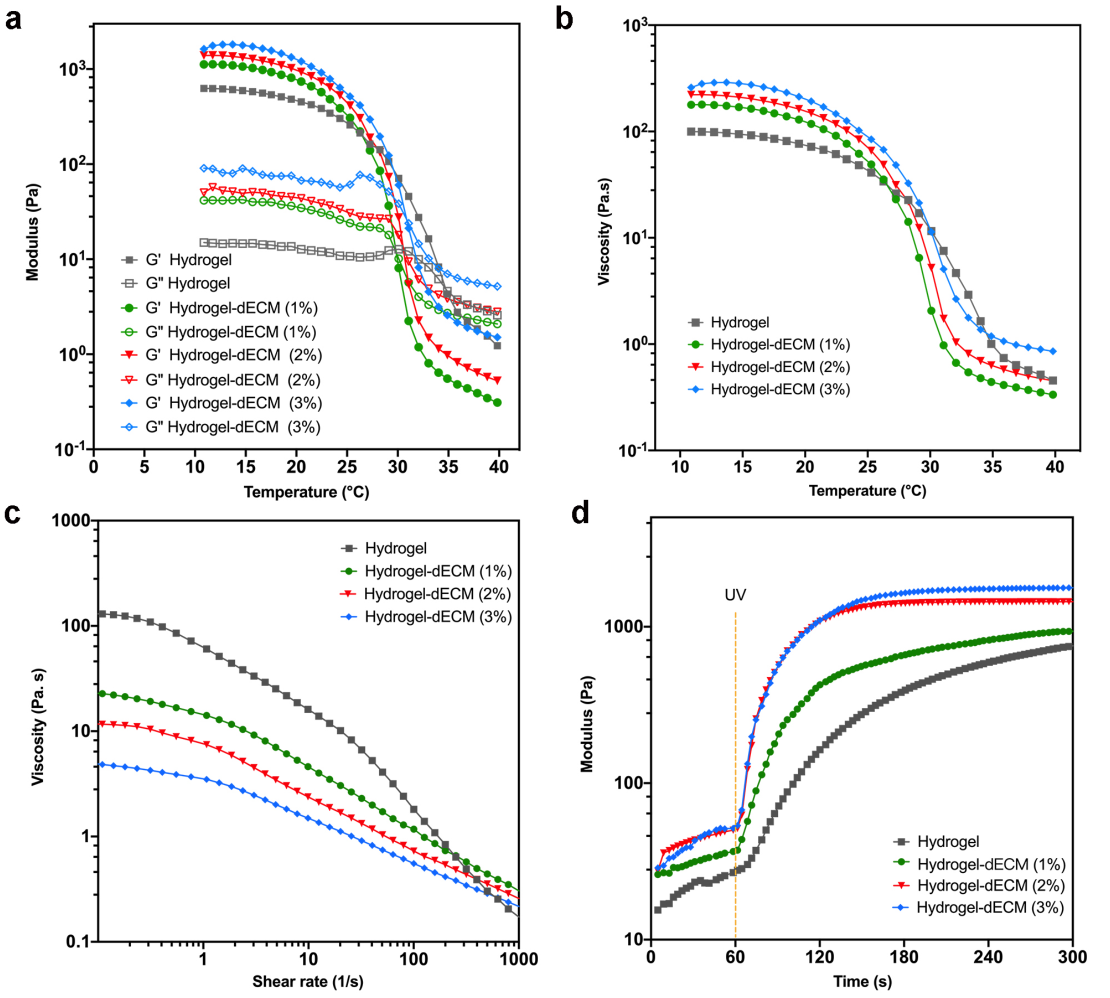

Figure S3. Evaluation of the rheological properties. **a,** Variation in dynamic storage (G′) and loss (G″) in a temperature amplitude sweep test, where the cross-over points between the gel and sol state (G′ = G″) represent gel–sol transition temperature. **b,** Viscosity during temperature amplitude sweep test. **c,** Viscosity measurement of different bioinks in the shear rate and dynamic frequency sweeps. **d,** Time-sweep of bioinks upon ultraviolet (UV) light irradiation (at 60 s).

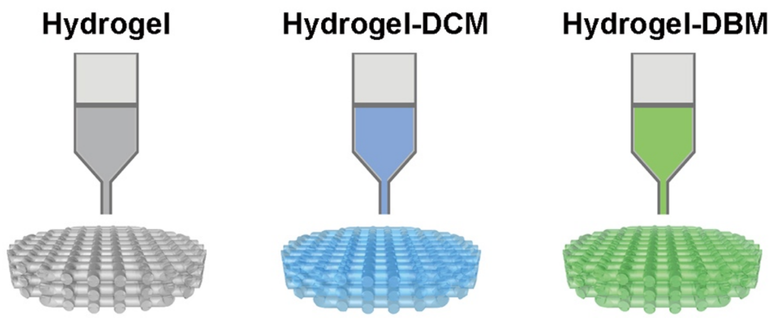

Figure S4. Illustration of 3D printing of Hydrogel, Hydrogel-DCM, and Hydrogel-DBM scaffolds.

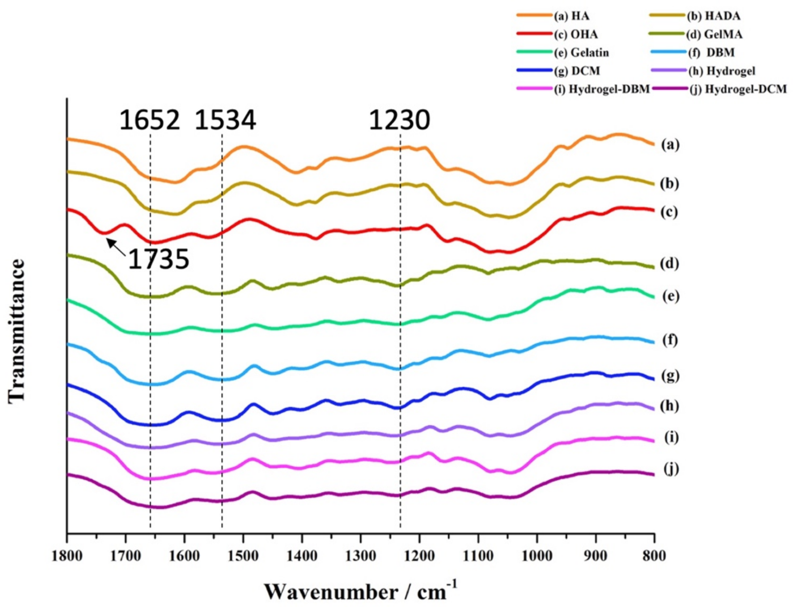

Figure S5. FTIR of different biomaterials and scaffolds.

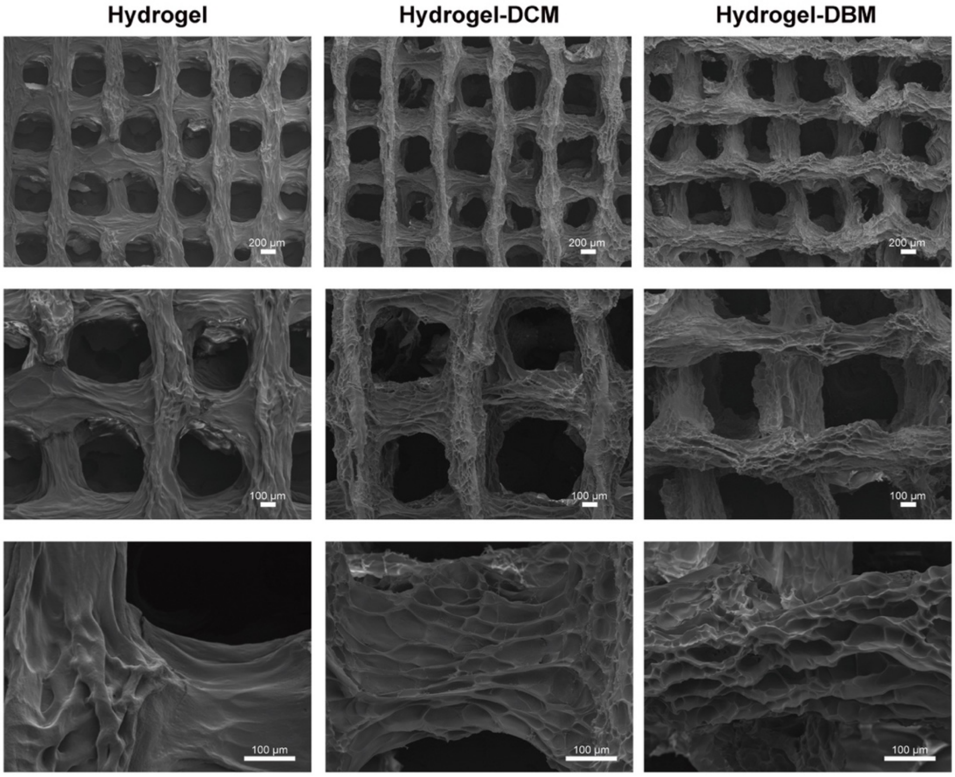

Figure S6. SEM images of the printed Hydrogel, Hydrogel-DCM, and Hydrogel-DBM scaffolds.

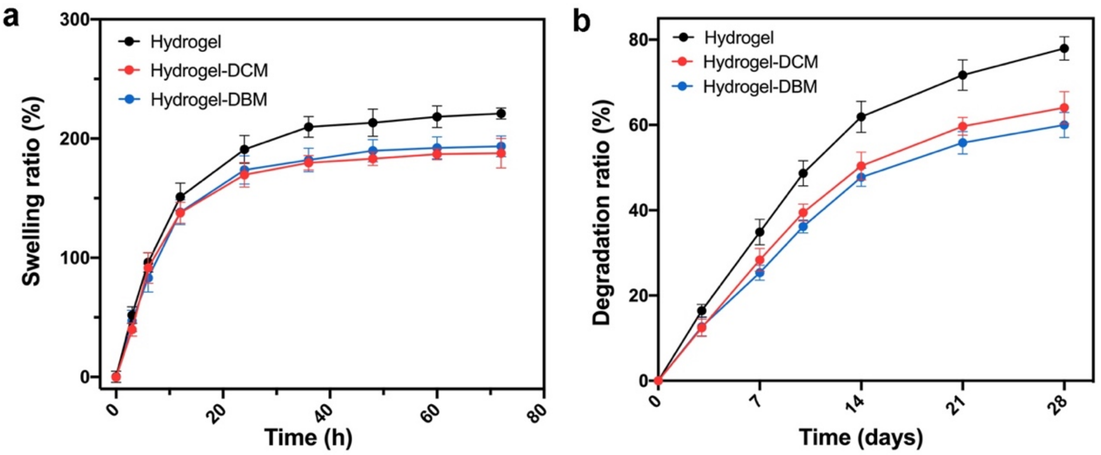

Figure S7. Dynamic swelling ratio (**a**) and degradation ratio (**b**) of the printed scaffolds.

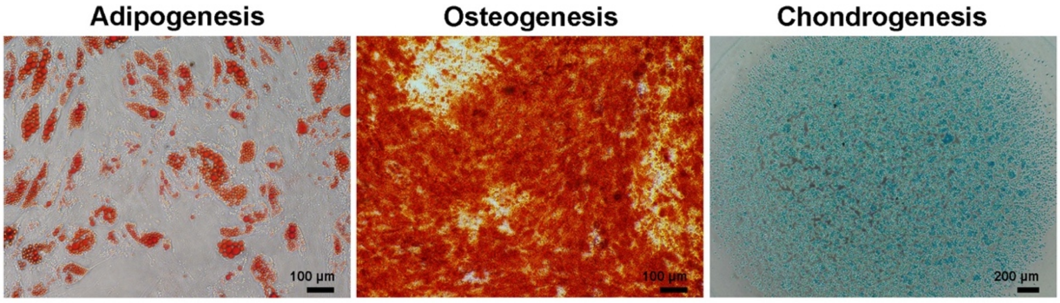

Figure S8. Multi-lineage differentiation potential of hADSCs identified by oil red O, alizarin red, and alcian blue staining.

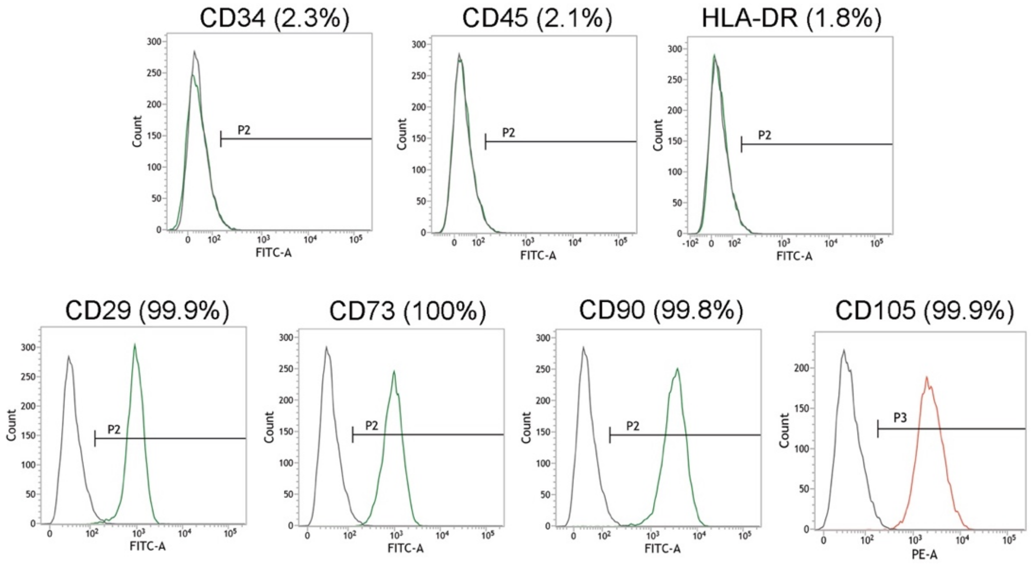

Figure S9. The detection of surface markers from hADSCs by flow cytometry.

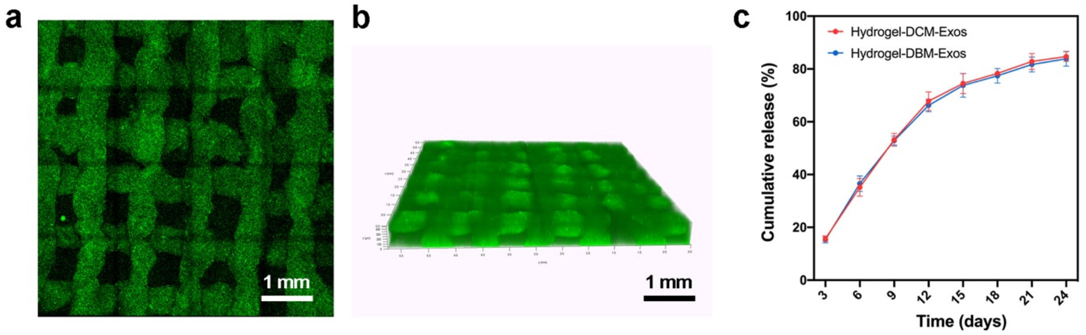

Figure S10. The distribution and release profile of exosomes. **a-b**, Exosomes (green) distributed evenly in the printed Hydrogel-DCM-Exos scaffold. **c**, Cumulative release profile of exosomes from the printed Hydrogel-DCM-Exos and Hydrogel-DBM-Exos hydrogel scaffolds over three-week period.

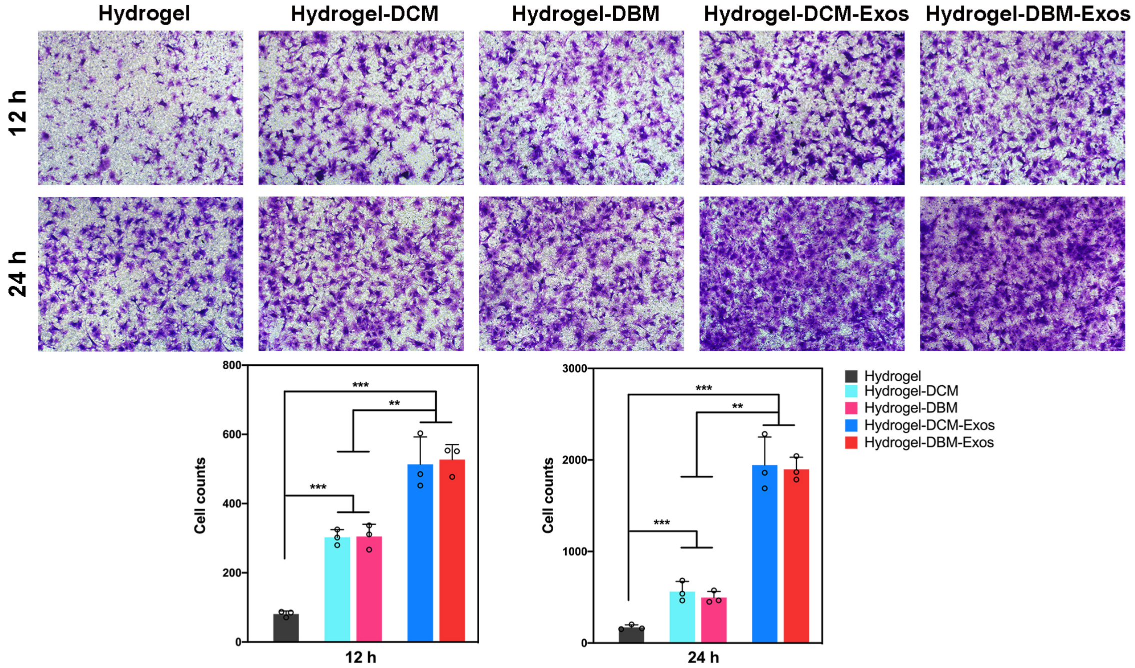

Figure S11. Co-culture of rBMSCs (upper chamber) and different scaffolds (lower chamber) by traswell assay at 12 and 24 h. $n = 3$ independent biological replicates, ** $P < 0.01$, *** $P < 0.001$.

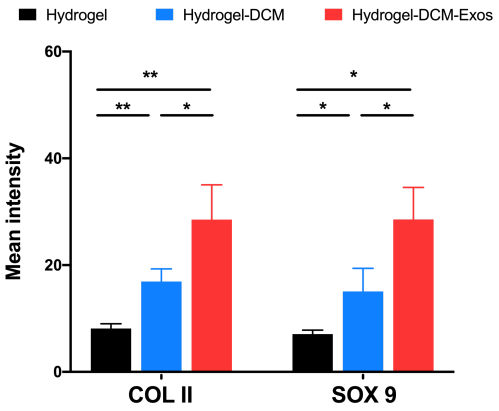

Figure S12. Quantitative analysis of the mean fluorescence intensities of COL II and SOX9 expression in rBMSCs grown in different scaffolds after incubated for 14 days. n = 3 independent biological replicates, * $P < 0.05$, ** $P < 0.01$.

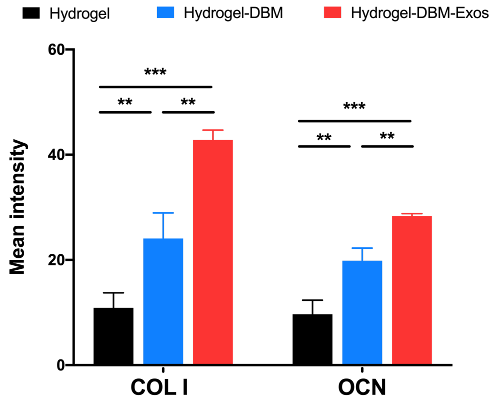

Figure S13. Statistical analysis of the mean fluorescence intensities of COL I and OCN expression in rBMSCs grown in different scaffolds after incubated for 14 days. n = 3 independent biological replicates, * $P < 0.05$, ** $P < 0.01$, *** $P < 0.001$.

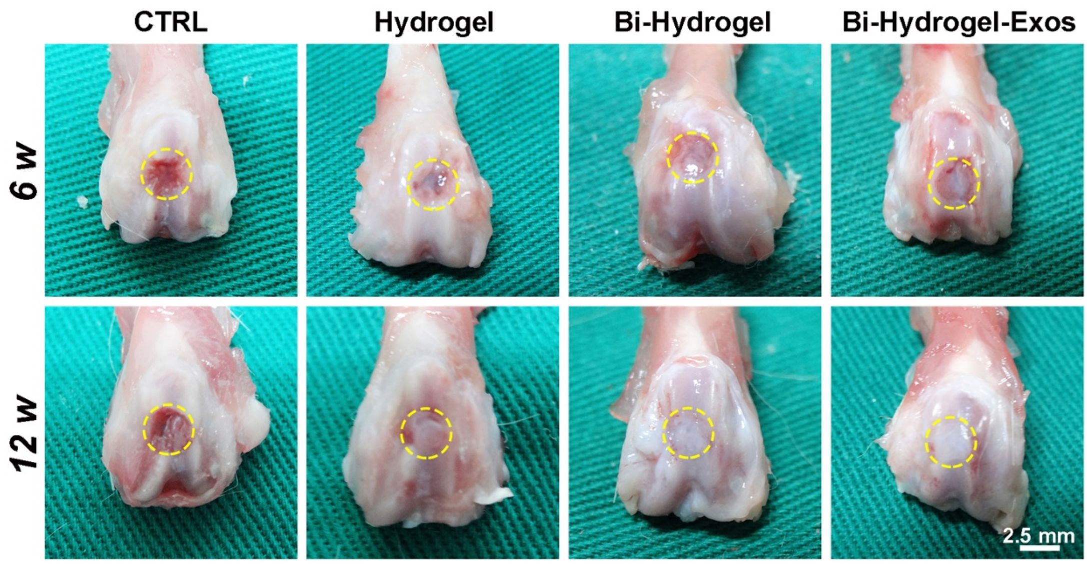

Figure S14. Macroscopic photographs of regenerated tissues in knee joints at 6 and 12 weeks post-operation. The circles indicate the boundary of defects. Scale bar = 2.5 mm.

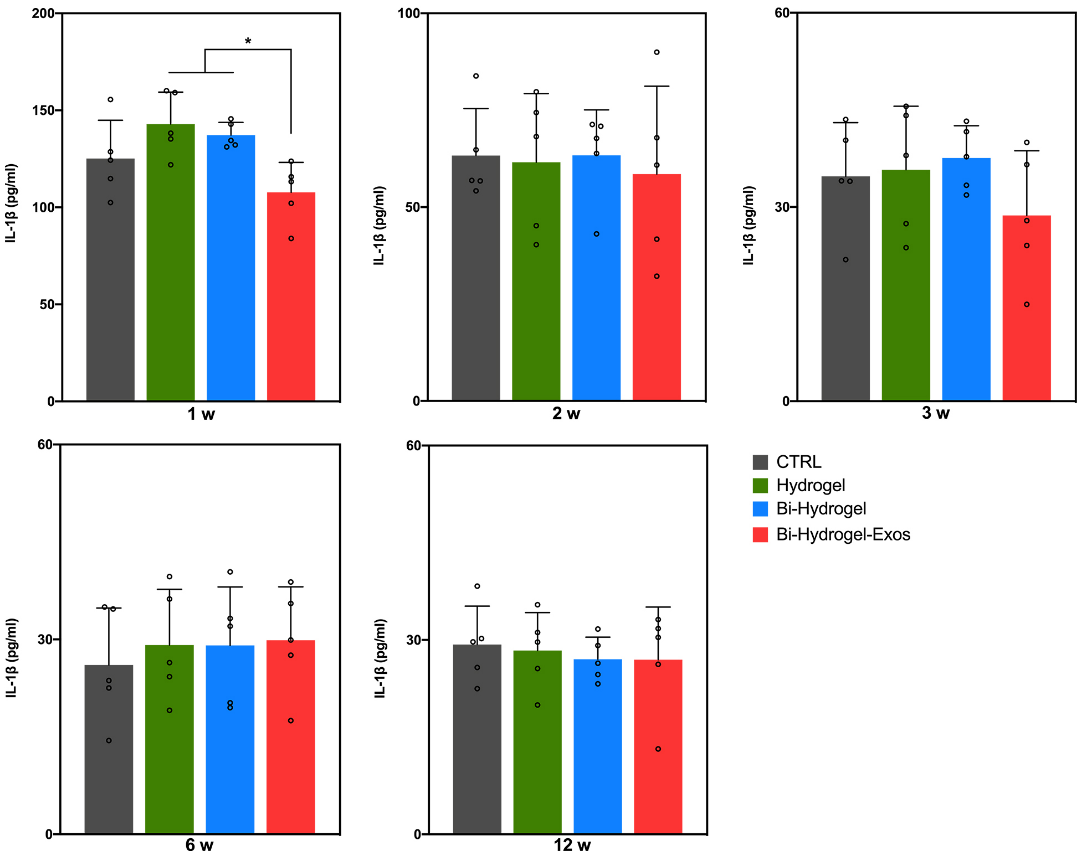

Figure S15. 1L-1β level in the serum of experimental animals at 1, 2, 3, 6, and 12 weeks after surgery. n = 5 individual rats, *P<0.05.

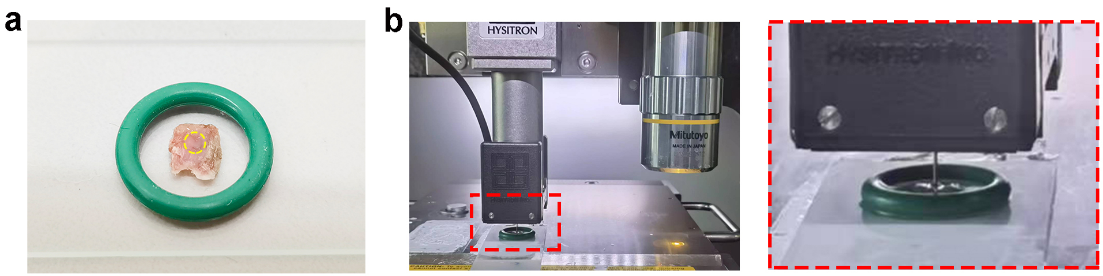

Figure S16. Nanoindentation assessment. **a,** Regenerated catilage tissue (marked by the circle) was collected and placed in the central area of a rubber ring to fill with PBS for hydration. **b,** Nanomechanical test was detected by the TI 950 TriboIndenter In-Situ system.

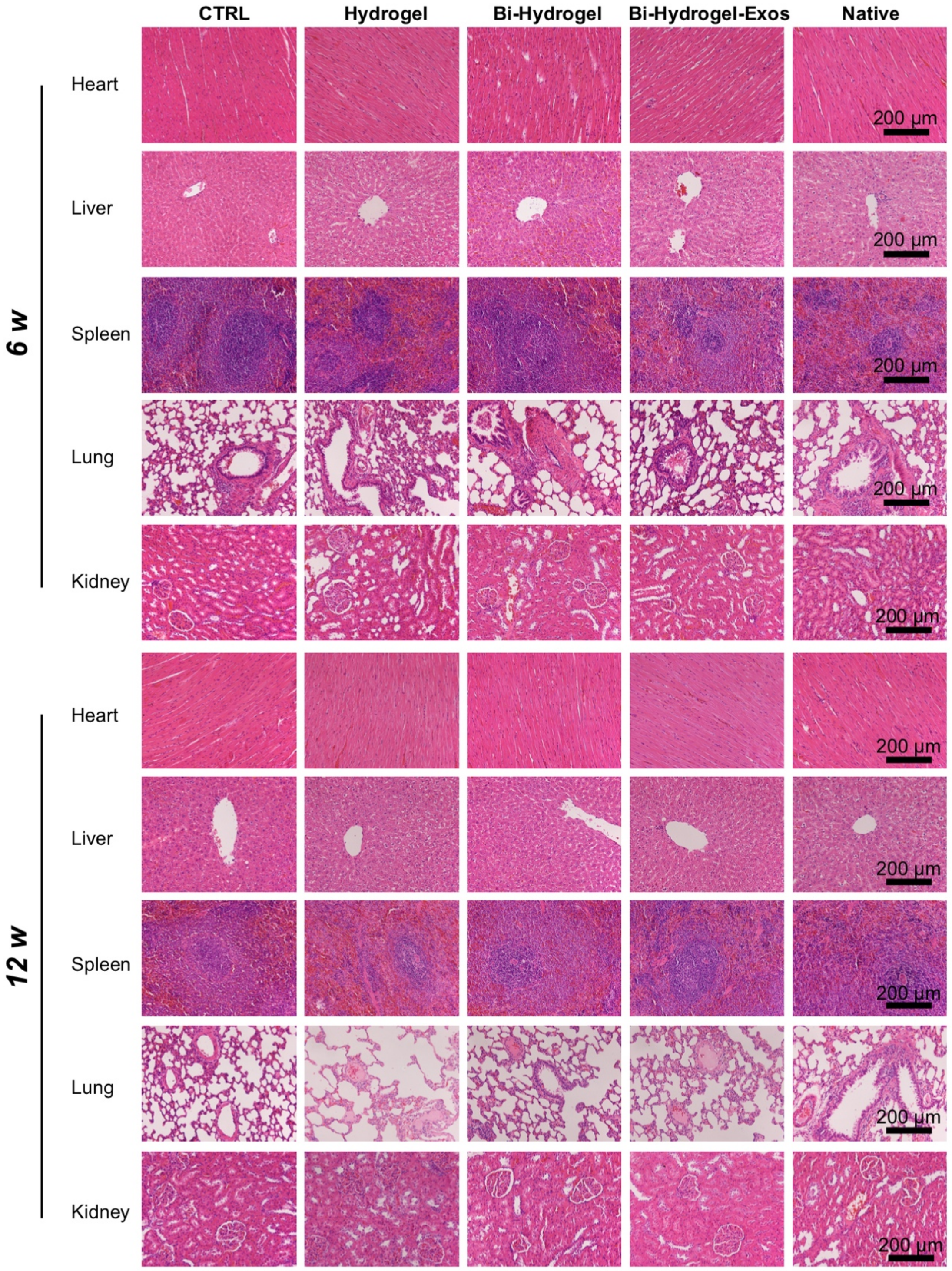

Figure S17. H&E staining of heart, liver, spleen, lung, and kidney tissues for different groups at 6 and 12 weeks post-operation. Scale bar = 200 μm.

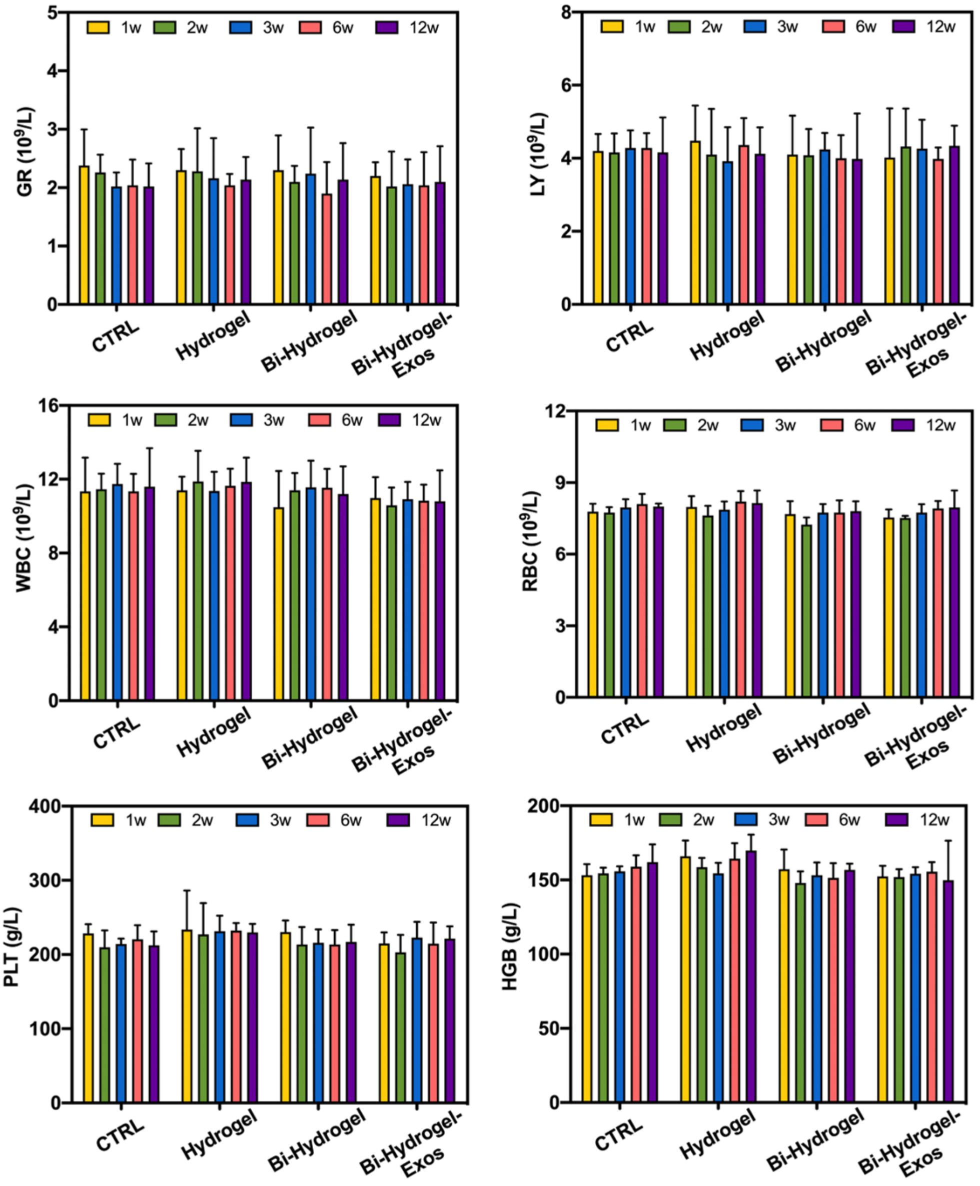

Figure S18. In vivo safety study. Hematology of neutrophil granulocytes (GR), lymphocytes (LY), white blood cells (WBC), red blood cells (RBC), platelets (PLT) and hemoglobin (HGB) at 1, 2, 3, 6, and 12 weeks after surgery. n = 5 individual rats.

Table S1. Primer information for quantitative real-time polymerase chain reaction.

Gene	Forward sequences (5'~3')	Reverse sequences (5'~3')
Acan	CATTCGCACGGGAGCAGCCA	TGGGGTCCGTGGGCTCACAA
Sox9	TCCCCGCAACAGATCTCCTA	AGCTGTGTGTAGACGGGTTG
Col II	CACCGCTAACGTCCAGATGAC	GGAAGGCGTGAGGTCTTCTGT
Col X	AACAAGGAAGGCACACAACC	AGCTGGGCCAATATCTCCTT
Col I	GCAATGCTGAATCGTCCCAC	CAGCACAGGCCCTCAAAAAC
Alpl	CCTTGAAAAATGCCCTGAAA	CTTGGAGAGAGCCACAAAGG
Ocn	CCGTTTAGGGCATGTGTTGC	CCGTCCATACTTTCGAGGCA
Runx2	GAGCACAAACATGGCTGAGA	TGGAGATGTTGCTCTGTTCG
Rn18s*	GTAACCCGTTGAACCCCATT	CCATCCAATCGGTAGTAGCG

* 18S ribosomal RNA (Rn18s) was used for normalization of gene expression.

Table S2. ICRS macroscopic evaluation of cartilage repair

Categories	Points
Degree of defect repair	
In level with surrounding cartilage	4
75% repair of defect depth	3
50% repair of defect depth	2
25% repair of defect depth	1
0% repair of defect depth	0
Integration to border zone	
Complete integration with surrounding cartilage	4
Demarcating border < 1 mm	3
3/4 of graft integrated, 1/4 with a notable border > 1 mm width	2
1/2 of graft integrated with surrounding cartilage, 1/2 with a notable border > 1 mm	1
From no contact to 1/4 of graft integrated with surrounding cartilage	0
Macroscopic appearance	
Intact smooth surface	4
Fibrillated surface	3
Small, scattered fissures of cracks	2
Several, small or few but large fissures	1
Total degeneration of grafted area	0
Overall repair assessment	
Grade I: normal	12
Grade II: nearly normal	8-11
Grade III: abnormal	4-7
Grade IV: severely abnormal	1-3

Table S3. Histological scoring system of cartilage repair

Categories	Points
Cell morphology	
Hyaline cartilage	4
Mostly hyaline cartilage	3
Mostly fibrocartilage	2
Mostly non-cartilage	1
Non-cartilage only	0
Matrix staining	
Normal	3
Slightly reduced	2
Markedly reduced	1
No metachromatic stain	0
Surface regularity	
Smooth (>3/4)	3
Moderate (>1/2–3/4)	2
Irregular (1/4–1/2)	1
Severely irregular (<1/4)	0
Thickness of cartilage	
>2/3	2
1/3–2/3	1
<1/3	0
Integration of donor with host adjacent cartilage	
Both edge integrated	2
One edge integrated	1
Neither edge integrated	0
Total maximum	14

References

[1] Q. Li, H. Yu, M. Sun, P. Yang, X. Hu, Y. Ao, J. Cheng, *Acta Biomater.* **2021**, *125*, 253.

[2] Q. Li, F. Zhao, Z. Li, X. Duan, J. Cheng, J. Zhang, X. Fu, J. Zhang, Z. Shao, Q. Guo, X. Hu, Y. Ao, *Front. Cell. Dev. Biol.* **2020**, *8*, 694.

[3] G. L. Ying, N. Jiang, S. Maharjan, Y. X. Yin, R. R. Chai, X. Cao, J. Z. Yang, A. K. Miri, S. Hassan, Y. S. Zhang, *Adv. Mater.* **2018**, *30*, e1805460.

[4] F. Gao, Z. Xu, Q. Liang, H. Li, L. Peng, M. Wu, X. Zhao, X. Cui, C. Ruan, W. Liu, *Adv. Sci.* **2019**, *6*, 1900867.

[5] H. Tan, C. R. Chu, K. A. Payne, K. G. Marra, *Biomaterials* **2009**, *30*, 2499.

[6] J. Chi, A. Li, M. Zou, S. Wang, C. Liu, R. Hu, Z. Jiang, W. Liu, R. Sun, B. Han, *Int. J. Biol. Macromol.* **2022**, *203*, 492.

[7] Y. Liang, X. Zhao, T. Hu, B. Chen, Z. Yin, P. X. Ma, B. Guo, *Small* **2019**, *15*, e1900046.

[8] F. Pati, J. Jang, D. H. Ha, S. Won Kim, J. W. Rhie, J. H. Shim, D. H. Kim, D. W. Cho, *Nat. Commun.* **2014**, *5*, 3935.

[9] A. N. Rindone, B. Kachniarz, C. C. Achebe, R. C. Riddle, A. N. O'Sullivan, A. H. Dorafshar, W. L. Grayson, *Adv. Healthcare Mater.* **2019**, *8*, e1801565.

[10] M. Sun, J. Cheng, J. Zhang, N. Wu, F. Zhao, Z. Li, H. Yu, X. Duan, X. Fu, X. Hu, H. Chen, Y. Ao, *ACS Biomater Sci Eng* **2021**, *7*, 916.

[11] C. Wang, M. Wang, T. Xu, X. Zhang, C. Lin, W. Gao, H. Xu, B. Lei, C. Mao, *Theranostics* **2019**, *9*, 65.

[12] S. Jin, Y. Wang, X. Wu, Z. Li, L. Zhu, Y. Niu, Y. Zhou, Y. Liu, *Adv. Mater.* **2023**, DOI: 10.1002/adma.202211602e2211602.
